# Overexpression of an AP2/ERF Type Transcription Factor *OsEREBP1* Confers Biotic and Abiotic Stress Tolerance in Rice

**DOI:** 10.1371/journal.pone.0127831

**Published:** 2015-06-02

**Authors:** V. Jisha, Lavanya Dampanaboina, Jyothilakshmi Vadassery, Axel Mithöfer, Saivishnupriya Kappara, Rajeshwari Ramanan

**Affiliations:** 1 Centre for Cellular and Molecular Biology, Hyderabad, India; 2 Max Planck Institute for Chemical Ecology, Department Bioorganic Chemistry, Jena, Germany; University of Delhi South Campus, INDIA

## Abstract

AP2/ERF–type transcription factors regulate important functions of plant growth and development as well as responses to environmental stimuli. A rice AP2/ERF transcription factor, *OsEREBP1* is a downstream component of a signal transduction pathway in a specific interaction between rice (*Oryza sativa*) and its bacterial pathogen, Xoo (*Xanthomonas oryzae* pv. *oryzae*). Constitutive expression of *OsEREBP1* in rice driven by maize *ubiquitin* promoter did not affect normal plant growth. Microarray analysis revealed that over expression of *OsEREBP1* caused increased expression of lipid metabolism related genes such as lipase and chloroplastic lipoxygenase as well as several genes related to jasmonate and abscisic acid biosynthesis. PR genes, transcription regulators and *Aldhs* (alcohol dehydrogenases) implicated in abiotic stress and submergence tolerance were also upregulated in transgenic plants. Transgenic plants showed increase in endogenous levels of α-linolenate, several jasmonate derivatives and abscisic acid but not salicylic acid. Soluble modified GFP (SmGFP)-tagged OsEREBP1 was localized to plastid nucleoids. Comparative analysis of non-transgenic and *OsEREBP1* overexpressing genotypes revealed that *OsEREBP1* attenuates disease caused by Xoo and confers drought and submergence tolerance in transgenic rice. Our results suggest that constitutive expression of *OsEREBP1* activates the jasmonate and abscisic acid signalling pathways thereby priming the rice plants for enhanced survival under abiotic or biotic stress conditions. *OsEREBP1* is thus, a good candidate gene for engineering plants for multiple stress tolerance.

## Introduction

Plants being sessile have evolved adaptive mechanisms to cope with various types of environmental stresses. Transcription factors are important candidates for adapting plants to stress tolerance since they regulate the expression of a number of stress responsive genes (referred to as regulons) by binding to specific *cis*-elements present in their promoters. A collection of >2000 known and predicted rice (*Oryza sativa*) transcription factors distributed in 63 families are available in the Database of Rice Transcriptional Factors [1]. Regulons regulated by AP2/ERF (Apetala2/ethylene Responsive factor), ABF/AREB (Abscisic acid binding factor/Abscisic acid responsive element binding) and NAC (NAM, ATAF and CUC domain containing) class of transcription factors are generally involved in abiotic stress responses [2]. Biotic stress responses depend on compatible or incompatible interactions between the pathogen and the host, leading to susceptibility or resistance in the plant via specific induction of transcription factors such as WRKY-type in response to pathogens [3].

The plant specific AP2/ERF (Apetala2/ethylene responsive factor) class of transcription factors are a large family with ~163 members in rice that regulate important functions of responses to environmental stimuli or plant growth and development depending on the presence of one or two highly conserved 60 amino acid AP2 domain in the protein [4, 5]. They regulate the expression of genes having a GCC box in their promoters, which include the DRE/CRT (drought responsive/C-Repeat) elements binding to CBF/DREB factors involved in abiotic stress responses. Overexpression of a tobacco (*Nicotiana tabacum*) AP2/ EREBP (Apetala2/ethylene responsive element binding protein) type transcription factor was shown to enhance biotic and osmotic stress tolerance in tobacco [6]. Five different DREBs {drought responsive element binding (*DREB1A*,*B*,*C*,*D* and *2A*)} have been cloned from rice and overexpression of rice *DREB1A* in *Arabidopsis thaliana* induced the expression of target stress inducible genes, resulting in increased tolerance of transgenic plants to drought, salt and cold tolerance [7]. *Arabidopsis* and rice constitutively overexpressing *DREB1A* showed tolerance to drought and cold but were compromised for growth, whereas use of a stress inducible promoter of *Rd29* for overexpression led to stress tolerance without stunting plant growth [8]. *AthDREB2A* full length clone neither affected the growth nor did it confer drought or salt tolerance when overexpressed in *Arabidopsis*, while a deletion of 135 to 165 residues transformed *DREB2A* into a constitutive form in the transgenic plants, which showed significant tolerance to drought [9]. Three of the four rice genes [(*OsBIERF 1–4*){(Oryza sativa benzothiadiazole (BTH)-induced ethylene responsive transcriptional factors (ERF)] with a single conserved ERF domain were found to be upregulated by salt, cold, drought, wounding as well as in an incompatible interaction between rice and fungal pathogen suggesting their role in biotic and abiotic stress [10]. Expression of *OsEBP2* (Oryza sativa ethylene-responsive-element binding protein 2) was induced in response to compatible interaction between rice and blast fungus and transiently induced by treatments with MeJA, ABA and ET indicating its possible role in abiotic stress [11]. An AP2/ERF like transcription factor Ath hardy (*Hrd*), when expressed in rice conferred drought tolerance with improved water-use efficiency and also increased shoot and root biomass [12]. Constitutive expression of *OsAP37* enhanced drought tolerance of rice in vegetative stage and increased grain yield under drought conditions [13]. The *Sub1A* (Submergence1A) gene which encodes for AP2 domain transcription factor enhanced survival in submergence tolerant rice cultivars by inhibiting shoot elongation [14]. Over expression of a tomato (*Lycopersicon esculentum*) ERF improved osmotic and drought tolerance in rice [15], while overexpression of *AthDREB1A* improved drought tolerance and *OsDREB1B* conferred salt tolerance in *indica* varieties of rice [16].

An AP2/ERF class of transcription factor *OsEREBP1* was identified as a downstream component of a signal transduction pathway in a specific interaction between rice and its bacterial pathogen, *Xanthomonas oryzae* pv. *oryzae* (Xoo) in yeast two-hybrid screens (unpublished data). The resistance gene *Xa21* in rice encodes a LRR- receptor kinase (LRK) protein which specifically recognizes a sulphated peptide Ax21, secreted via the Type I secretion system of Xoo strains [17, 18], resulting in an incompatible interaction between the host and the pathogen [19]. The extracellular LRR domain of Xa21 specifically recognizes the Ax21 ligand from Xoo and results in the autophosphorylation of the serine/threonine residues in the intracellular kinase domain [20] that triggers a signal transduction pathway leading to resistance by the activation of defense genes. The intracellular kinase domain of Xa21 including the JM region (juxtamembrane, a portion of cytoplasmic domain between the transmembrane and kinase region) termed as Xa21K668 [21] interacts with downstream proteins in the signaling pathway. Several proteins interacting with Xa21K668 *in vitro* were identified using the yeast-two hybrid system and these have been referred to as Xa21-binding proteins (Xbs). One of these Xbs, Xb22 was further used as bait in yeast-two hybrid screens to identify Xb-interacting partners. Two proteins were found to interact with Xb22 and one of them was an AP2-EREBF transcription factor (*OsEREBP1*), which was shown to be a positive regulator of resistance to Xoo infection in phenotypic assays [22]. This study elucidates the role of *OsEREBP1* transcription factor in various stress responses of rice.

## Materials and Methods

### Plasmid construction for overexpression and rice transformation

The full length coding sequence was PCR-amplified using OsEREBP1FL primers (S1 Table), from a cDNA library of japonica rice cultivar prepared from seedlings inoculated with the blast fungus *Magnaporthe griseae* and cloned into the pENTR gateway vector (Invitrogen) as per the supplier’s manual, to obtain the AP2/pENTR plasmid. For overexpression in japonica cv. Kitaake, the *OsEREBP1* cDNA in pENTR/TOPO was recombined into the Ubi-NC1300-Rfa vector using Gateway LR Clonase (Invitrogen) to obtain AP2/pNC1300 plasmid [23]. Ubi-NC1300-Rfa is a derivative of pC1300 with maize ubiquitin promoter [24] and nopaline synthase to which Gateway cassette has been added. The construct was then mobilized into *Agrobacterium* strain EHA105 for transformation of rice calli and transformed calli were selected for hygromycin resistance as described [24]. Shoots were regenerated from the hygromycin resistant calli and induced for root formation. The regenerated plants were transferred to soil and allowed to set seeds. The T3 stable transgenic rice plants were obtained.

### Microarray analysis

RNA from fully grown third leaf of transgenic and non-transgenic plants at the four-leaf stage were used for hybridization of Rice Affymetrix gene-chip (51K arrays) containing probe sets designed from 48564 japonica and 10260 indica gene sequences according to the Affymetrix GeneChip expression analysis technical manual. Three biological replicates were used for the experiment. Annotation of the differentially expressed probes was done using NetAffyx software of Affymetrix and further validated using BLASTX search through NCBI.

### PCR analysis

Total RNA was extracted from fully grown leaf of transgenic (4–3 and 2–4) and non-transformed four week old rice seedlings using trizol reagent (Invitrogen) and treated with RNase-free DNase I (Invitrogen) to prevent genomic DNA contamination. First-strand cDNA was synthesized from 5μg of total RNA from each sample using superscript-III RT-PCR kit (Invitrogen) and oligo–dT 18 mer primer and stored at -20°C for further use.

Regular PCR was performed in a PTC-200 Peltier Thermal cycler (MJ Research) with a 20 μl reaction mixture containing 1 μl template, 5 pmoles of each primer. 200 μM of each of the four deoxynucleotide phosphates and 1 unit Taq polymerase (Bangalore Genie, India). The PCR conditions were 94°C for 5 min for denaturation 30 cycles of 94°C for 30 sec, 55°C for 30 sec and 72°C for 1 min followed by final extension of 72°C for 10 min.

All qRT-PCR was performed on an ABI prism 7900 HT sequence detection system (Applied Biosystems). A typical 10 μl reaction mixture contained 5 μl of 2X SYBR green dye (Invitrogen), 1 pmoles of each primer, 1 μl of template and 2 μl of distilled water. All reactions were set up in triplicates, in a 386-well optical reaction plate. The PCR conditions were as follows: 95°C for 10 min,: 95°C for 15 sec, 60°C for 30 sec and 72°C for 30 sec for 40 cycles. The levels of *OsActin1* served to normalize the expression ratio for each gene and the changes in expression were calculated by the ΔΔCt method [25]. To calculate the relative expression of a given gene, all expression values were normalized to that of Kitakee which was taken as calibrator and its expression value was adjusted to one. The values reported are the mean of at least three independent experiments (three biological replicates) with each sample analyzed in triplicate. Primers for real time PCR are listed in S1 Table.

### Accession numbers

The microarray data has been submitted to the GEO repository and assigned GEO accession number GSE46173 and can be viewed at http://www.ncbi.nlm.nih.gov/geo/query/acc.cgi?acc=GSE46173. Sequence data from this article can be found in the Michigan State University Rice Genome Annotation Project database (http://rice.plantbiology.msu.edu) under the following accession numbers: *Jmt* (LOC_Os05g01140), *PR10* (LOC_Os12g36830), *NAC6* (LOC_Os03g60080), *PBZ1* (LOC_Os12g36850), *Wrky62* (LOC_Os09g25070), *OsEREBP1* (LOC_Os02g54160), *LOX* (LOC_Os12g37320), *OsLis* (LOC_Os02g02930), *Actin* (LOC_Os03g50885), *Xb22* (LOC_Os07g07540).

### Xb22a-smGFP and OsEREBP1-smGFP plasmid construction and transient expressions in rice protoplast

The coding regions of Xb22a and OsEREBP1 without the stop codon, were PCR amplified using OsEREBP1-smGFP and Xb22a-smGFP forward and reverse primers (S1 Table) and the amplified product was cloned into pENTR/D-TOPO/D vector (Invitrogen). The positive clones were sequence confirmed and the inserts were recombined in-frame into the coding region of soluble-modified green fluorescent protein (smGFP) of pUbi-smGFP binary vector [26] using Gateway LR Clonase (Invitrogen). Protoplasts were isolated from stem and sheath of rice seedlings and transient expressions of smGFP constructs were performed by introducing plasmids into rice protoplasts using the PEG-mediated transformation method [27]. Images were collected through 60x (numerical aperture = 1.40) oil immersion lens of Leica TCS SP5 confocal microscope (Leica Microsystems, Weitlzar, Germany) with argon laser excitation wavelength of 488nm and detection windows of 500–530nm and 640–726nm for GFP and chlorophyll autofluorescence, respectively. All fluorescent experiments were independently performed at least three times.

### Estimation of total lipids and phytohormones

Total lipids were extracted from the leaves of 2–3 week old greenhouse grown plants according to Griffiths *et al*., [28]. Young leaves (~0.2g fresh weight) were homogenized with a pestle and mortar containing 0.15M acetic acid (1ml) and chloroform/methanol (1:2 v/v; 7.5ml) for about 2min and transferred to conical glass centrifuge tubes. Samples were centrifuged at low speed and the lower chloroform phase containing the lipids were removed to glass vials and evaporated under nitrogen. All steps were done in dim light with chilled solvents. Lipids were quantified by GC-MS analysis as their fatty acid methyl ester derivatives after transmethylation with 2.5%v/v H_2_SO_4_ in anhydrous methanol (2ml) followed by hexane extractions. Extracts were evaporated under N_2_ and further redissolved in 200μl of hexane. 3μl of sample is injected in to the column along with the internal standard nonadecanoic acid. GC-MS system used was of Agilent Technologies equipped with thermal conductivity detector (TCD). Method used was GCMS-FAMES M and separation was achieved on a HP-5MS column with helium as the carrier gas at the rate of 1.3ml/min.

For phytohormone analysis, finely ground leaf material was extracted with 1.5 mL of methanol containing 60 ng of 9,10-D_2_-9,10-dihydrojasmonic acid, 60 ng D_4_-salicylic acid, 60 ng D_6_-abscisic acid (Santa Cruz Biotechnology), and 15 ng of jasmonic acid-[^13^C_6_]isoleucine conjugate as internal standards according to the method described by Vadassery et al. [29]. Phytohormones were quantified relative to the signal of their corresponding internal standards. The peak of the endogenous bioactive form of JA-Ile, (+)-7-*iso*-Jasmonoyl-*L*-isoleucine was used for JA-Ile quantification [30]. The authentic 12-OH-JA-Ile (*N*-((±)-tuberonoyl)-L-isoleucine was used for the determination of the response factor. 12-OH-JA-Ile was quantified on the MRM m/z 338/130 (DP -50V, CE -30V) using the signal of the internal standard, jasmonic acid-[^13^C_6_] isoleucine conjugate applying an experimentally determined response factor of 1.

### Inoculations with Xoo and disease scoring

BXO43 strain of Xoo, which is the most prevalent pathotype in India was inoculated in peptone-sucrose medium and incubated for 48 h at 28°C in a shaker incubator adjusted to 180 rpm. The culture was centrifuged at 5000 rpm in refrigerated centrifuge and the bacterial pellet was resuspended in the original volume of sterile distilled water. Two topmost fully expanded leaves in all the tillers of six week old rice seedlings were inoculated by clipping the leaves with a scissor dipped in inoculum [31]. The lesion lengths were measured in centimeters using a ruler at different time intervals from at least ten inoculated leaves for each rice sample and average was plotted as a graph with error bars for each recording. For Xoo colony counts from inoculated leaves, 10 cm of leaf tissue from the top, including lesions and tissue showing no lesions was collected at different time intervals and extracted in 5 ml water to harvest bacteria. The extract was diluted accordingly and plated out on peptone sucrose agar (PSA) plates containing 15 mg/l cyclohexamide (to avoid fungal contamination) to get single colonies. The colonies were counted and the bacterial population per leaf was calculated.

### Drought tolerance testing of transgenic plants

Seedlings grown in soil-filled, 15 cm diameter pots were saturated with water (20% W/W) and the soil surface was mulched with a 2 cm thick layer of polythene beads, for minimizing soil evaporation [32]. Eight seedlings per pot with two replicates for drought stress (DS1 and DS2) and one replicate for well watered (WW) control treatment were maintained by uniform watering till the beginning of the experiment in green house conditions. Drought was imposed at four leaf stage (3 weeks after germination) in the first experiment (DS1) and panicle initiation or booting stage (~8 weeks from germination) during second experiment (DS2). Regular watering was done to control WW pot while water was completely withheld in DS1 and DS2 treatments allowing gradual depletion and drying of pots till the plants showed intense drying symptoms and complete rolling of leaves leading to browning of leaf sheaths. Each of the pots was re-watered and the pattern of recovery was closely monitored and plants were scored as viable if new leaves appeared during recovery period.

### Submergence treatment

Two week old seedlings grown in soil-filled 15 cm diameter plastic pots were completely submerged in a plastic tub filled with 60 cm water and covered with polythene sheet in greenhouse conditions as described earlier [33]. The plants were removed after 5, 7 or 10 days of submergence, photographed and the leaf length was measured with a scale before and after submergence. Comparisons of the lengths were also made between plants grown under aerobic and submerged conditions. Plants were scored as viable if new leaves appeared upon desubmergence. The leaf tips were collected immediately after de-submergence for histochemical staining to detect reactive oxygen species according to Fitzgerald et al., [34] except that after incubation with DAB or NBT, leaves were cleared by soaking in 95% ethanol at 70°C for 15 minutes.

## Results

### Constitutive expression *OsEREBP1* in rice does not affect normal plant growth

**Fig 1:**
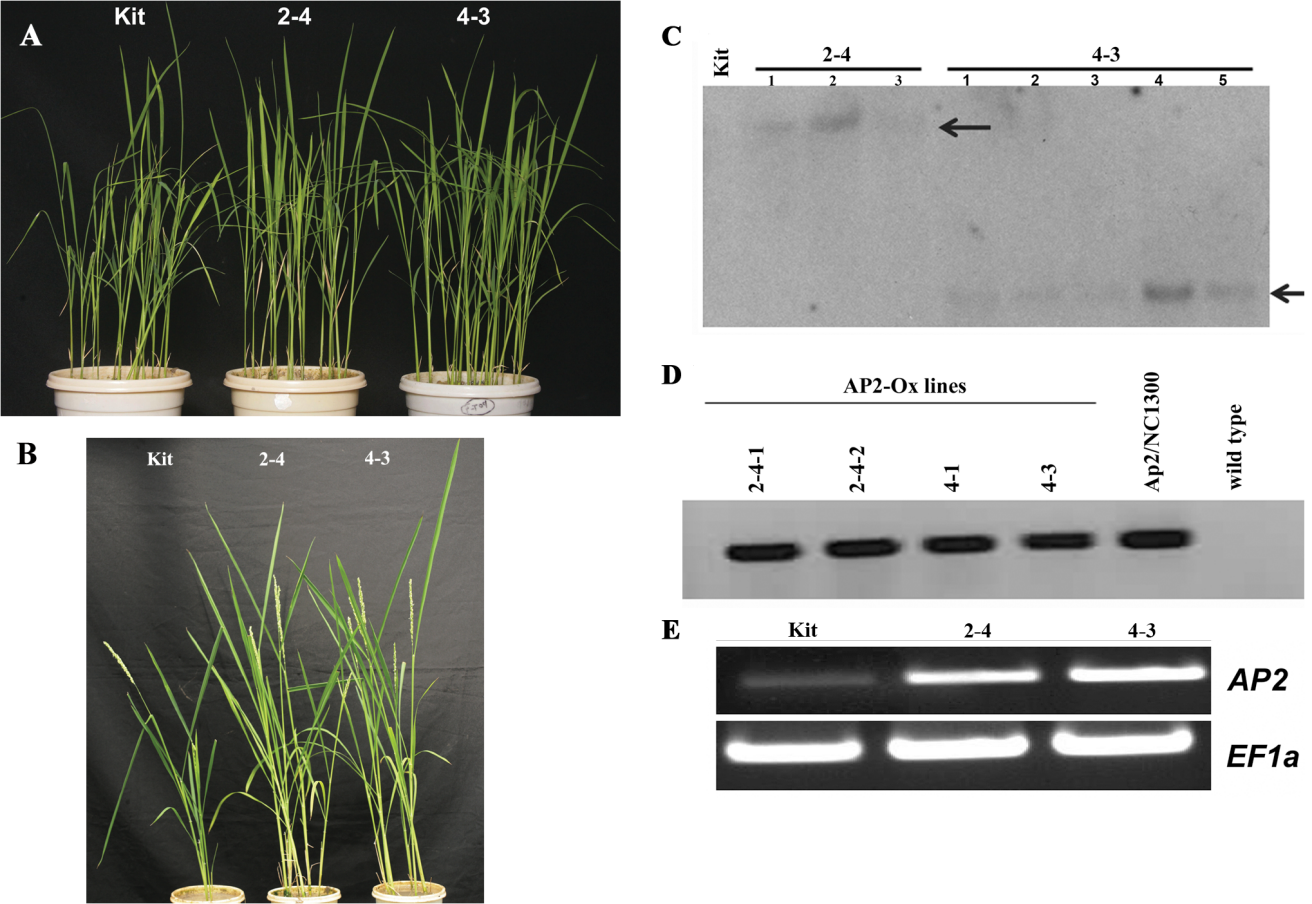
Phenotypes and genotypes of transgenic rice plants. A) Photographs were taken of six week-old seedlings grown under greenhouse conditions. B) Pictures were taken of plants after the panicle initiation stage. **C**) Southern blot hybridization of *BamH*I-digested genomic DNA showed single independent insertions in progenies of 2–4 and 4–3 (transgenic lines), whereas no band was observed in non-transformed Kitaake (control) when probed with hygromycin gene obtained by PCR amplification of pC1300 plasmid using *HygS/AS* primer pair. D) PCR analysis of genomic DNA using a vector-specific (*UbiS*) and insert-specific (*AP2AS*) primer pair showed presence of transgene in the progenies of 2–4 and 4–3 and not in Kitaake. Ap2/pNC1300 plasmid DNA was used as positive control. E) RT-PCR of cDNA using *OsEREBP1* primers showed increased transcript in 2–4 and 4–3 as compared to Kitaake. *EF1a* was used to normalize the cDNA. All primer details are given in S1 Table.

The full length cDNA of *OsEREBP1* was cloned in pENTR vector (AP2/pENTR) and was confirmed by sequencing. The *OsEREBP1* cDNA is comprised of 1098 bp and codes for 365 amino acid protein of about 40.1 kDa with a predicted pI of 4.89. The protein shows a highly conserved AP2 domain, a highly acidic N-terminal region and a putative nuclear localization signal (NLS). For overexpression of *OsEREBP1* in rice, AP2/pNC1300 plasmid was constructed in which the expression of full length cDNA was driven by maize ubiquitin (Ubi) promoter. Transgenic plants were successfully obtained following *Agrobacterium* mediated transformation of rice cultivar Kitaake. The transgenic plants did not show any undesirable growth phenotypes at seedling stage (Fig 1A) or at reproductive stages and produced fertile seeds (Fig 1B). Two independent transgenic lines were confirmed by Southern analysis to have single insertion of the transgene and were selected for further characterization and the presence of transgene in the progenies of the two stable T3 transformants of Kitaake variety of japonica rice was confirmed by PCR (Fig 1C and 1D). RT-PCR analysis of the transcript levels of *OsEREBP1* (also referred to as *AP2*) revealed that the transgenic lines had higher levels of transcript as compared to the wild type (Fig 1E).

### Activation of genes involved in jasmonates and abscisic acid biosynthetic pathways and defense responses in *OsEREBP1-ox* plants

**Fig 2:**
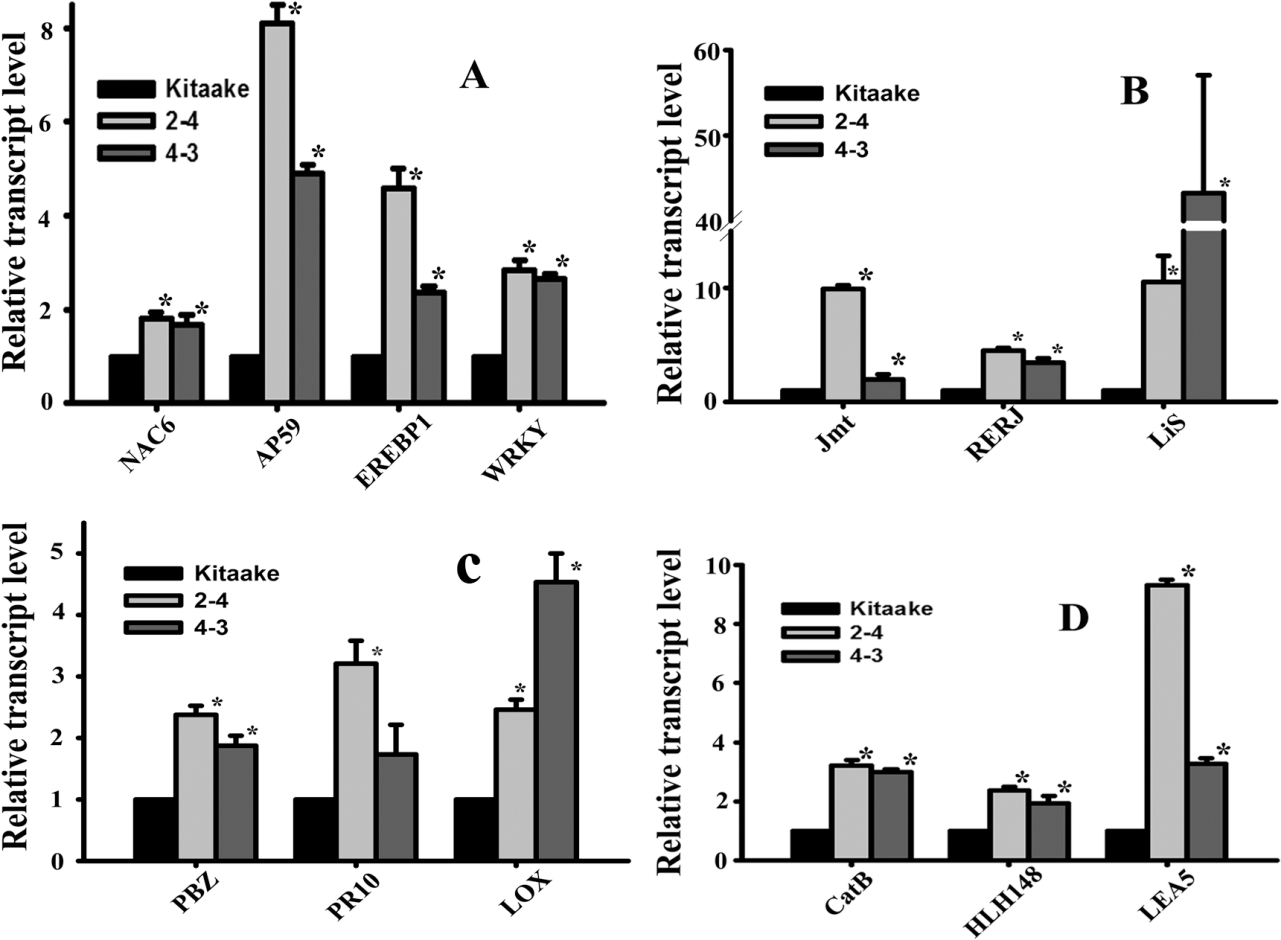
Transcript levels of genes showing upregulation in *OsEREBP1-ox* plants. Total RNA was extracted from 4 week old non-transformed (Kit) and transgenic (4–3 and 2–4) plants and qPCR was performed with primers for genes which include (A) *NAC6*, *AP59*, *EREBP1 & WRKY* (transcription factors); (B) *Jmt*, *RERJ & OsLis* (Jasmonate biosynthesis and regulation); (C) *PBZ*, *PR10*, *Lox* (Pathogenicity related); (D) *CatB*, *HLH148 & Lea5* (ABA pathway). *Actin* was used as internal standard. Primer details are given in S1 Table. Data representing mean±SE from three independent biological replicates was subjected to further statistical analysis by one way ANOVA using SigmaPlot Version 11.0. The asterix indicate *p* value of 0.05.

Global gene expression analysis of *OsEREBP1*-overexpressing (*OsEREBP1-ox*) transgenic line (4–3) versus non-transgenic control samples was carried out using the Affymetrix gene chip microarrays in order to identify molecular events associated with *OsEREBP1* overexpression. The CEL files generated by the Gene Chip Operating System (Affymetrix) were subjected to GeneSpring 12.6—GX—PA (Agilent Technology) for further analysis. The GC-RMA algorithm was used for data normalization. Genes showing differential expression were identified using array data from two of three independent experiments since hierarchical cluster analysis of microarray data from three biological replicates showed that two replicates grouped together while the third replicate formed a different cluster thereby lowering the significance (*P* value) of the fold-changes. Genes showing >1.5-fold difference with P value <0.05 were selected to obtain higher stringency in selection. The selected genes showed similar regulation between the untransformed Kitaake and 4–3 *OsEREBP1-ox* line grown under two different growth regimes, thus strengthening the confidence in the data. Among the thirteen genes validated by quantitative real time PCR (qPCR) analysis, all showed the same expression pattern as in microarray results (Fig 2).

A total of 2395 genes were differentially expressed in the overexpressor line which included 1348 up-regulated and 1047 down-regulated genes. Regulatory genes included transcription factors involved in stress responses such as four NAC-domain containing, fourteen AP2-domain containing including *OsEREBP1* and fifteen *WRKY*-type genes (Table 1).

**Table 1:** Genes up-regulated in *OsEREBP1-ox* plants.

Broad-Function Category	Gene Locus id	Gene name	Fold change
Transcription Factors	LOC_Os11g03300.1	(ONAC 122/131)	4.2
	LOC_Os03g60080.1	(OsNAC6)	2.9
	LOC_Os07g12340.1	(OsNAC3)	2.6
	LOC_Os01g60020.1	(OsNAC4)	1.9
	LOC_Os11g03540.1	AP2/EREBP131	7.9
	LOC_Os10g41330.2	DRE response factor 12	5.3
	LOC_Os02g43790.1	AP59	3.1
	LOC_Os01g54890.1	AP2/EREBP78	2.7
	LOC_Os04g52090.1	AP39	2.3
	LOC_Os08g31580.1	AP2/EREBP170 (PR)	1.7
	LOC_Os04g32620.1	AP2/EREBP89 (PR, DRE)	1.7
	LOC_Os03g08490.1	AP2/EREBP28	1.7
	LOC_Os08g31580.1	AP2/EREBP170 (PR)	1.7
	LOC_Os06g07030.1	AP2/EREBP142 (TINY-like)	1.6
	LOC_Os01g04800.1	AP2/EREBP129	1.6
	LOC_Os02g54160.1	OsEREBP1	1.5
	LOC_Os05g41780.1	AP2/EREBP51	1.5
	LOC_Os02g32140.1	AP2/EREBP15	1.5
	LOC_Os11g02530	OsWRKY40	4.6
	LOC_Os12g02450	OsWRKY64	4.6
	LOC_Os09g25070.1	OsWRKY62 (Xa21 binding)	3.9
	LOC_Os12g02440.1	OsWRKY95	3.5
	LOC_Os05g50610.2	OsWRKY8	3.4
	LOC_Os01g60640.1	OsWRKY21	2.6
	LOC_Os09g25060.1	OSWRKY76	2.3
	LOC_Os06g44010.1	OSWRKY28	2.3
	LOC_Os11g02480.1	OsWRKY46	2.1
	LOC_Os05g27730.1	OsWRKY53	2
	LOC_Os11g29870.1	OsWRKY72	2
	LOC_Os11g02540.1	OsWRKY50	1.9
	LOC_Os05g39720.1	OsWRKY70	1.8
	LOC_Os02g08440.1	OsWRKY71 (ABA)	1.7
	LOC_Os04g21950.1	OsWRKY51 (ABA)	1.6
Jasmonic acid biosynthesis	LOC_Os06g11290	12-oxophytodienoate reductase	3.7
	LOC_Os08g35740	12-oxophytodienoate reductase	1.5
	LOC_Os05g01140	jasmonate O-methyltransferase	2.1
	LOC_Os02g02930	OsLiS linalool synthase	9.9
	LOC_Os03g08999	Alcohol dehydrogenase	5.7
	LOC_Os12g05440	CYP94C	2.9
	LOC_Os12g14440	Jasmonate-induced protein	1.7
	LOC_Os04g23550	RERJ1	2.3
Pathogenicity related genes	LOC_Os12g36850	pathogenesis-related Bet v I family protein PBZ1 (PR10B)	6.7
	LOC_Os08g39840.1	LOX	8.9
	LOC_Os12g37320	LOX	3.3
	LOC_Os12g37260.1	LOX	1.5
	LOC_Os07g03730.1	SCP-like extracellular protein (PR1a)	18.6
	LOC_Os12g36880.1	pathogenesis-related Bet v I family protein PR10A	4.5
	LOC_Os12g36830.1	pathogenesis-related Bet v I family protein PR10	35.2
	LOC_Os03g28940	OsJAZ1	1.8
ABA biosynthesis and regulation	LOC_Os02g47510	9-cis-epoxycarotenoid dioxygenase 1, chloroplast (OsNced1)	3.9
	LOC_Os03g53020	OsbHLH148	1.7
	LOC_Os01g21250	LEA5	1.6
	LOC_Os06g51150	CatB	1.7

Genes that are >1.5-fold induced in *OsEREBP1-ox* plants relative to non-transgenic controls.

Biosynthesis of oxylipins like JA is initiated by the coordinated activities of lipases and lipoxygenases resulting in generation of unsaturated fatty acids. Interestingly, six lipases and four plastidic lipoxygenases and several genes involved in Jasmonic acid (JA) metabolism and regulation including OPDA reductase, JA-O methyl transferase (*Jmt*), *CYP94C1*, *OsJaz1* and linalool synthase (*OsLis*10-fold increase) and *RERJ1* (2.3-fold) were upregulated in transgenic plants. Transcript levels of *OsNCED1* coding for 9-cis-epoxycarotenoid dioxygenase, a key gene in abscisic acid (ABA) biosynthesis and *AP39* a transcription factor that upregulates *OsNECD1* causing increase in endogenous levels of ABA [35] were also elevated. qPCR results showed increased levels of *Jmt*, *RERJ1*, *OsLis* involved in JA metabolism and regulation as well as *AP59*, *CatB*, *HLH148* and *Lea5* involved in ABA pathway in 4–3 and 2–4 transgenic lines as compared to non-transgenic control (Fig 2).

As many as 93 defense response genes showed up-regulation in the *OsEREBP1-ox* lines compared to the untransformed wild type plants (S2 Table). The transcript levels of *WRKY62* (which interacts with Xb10 in the Xa21-mediated resistance pathway), *PR1b* and *PBZ1* genes which are specifically induced following bacterial infection were highly elevated indicating the role of *OsEREBP1* in bacterial resistance as confirmed by qPCR analysis (Fig 2). *Lox* gene, which is induced specifically in response to fungal infection and confers resistance against the fungal pathogen showed higher levels of expression in transgenic plants. Nine genes of the alcohol dehydrogenase family involved in alcohol fermentation during anaerobic conditions showed up-regulation (Table 1). These results suggest a role for *OsEREBP1* in bacterial, fungal, anaerobic and abiotic stress pathways by modulating JA and ABA phytohormones.

### *OsEREBP1* and its interacting protein are localized to chloroplast

**Fig 3:**
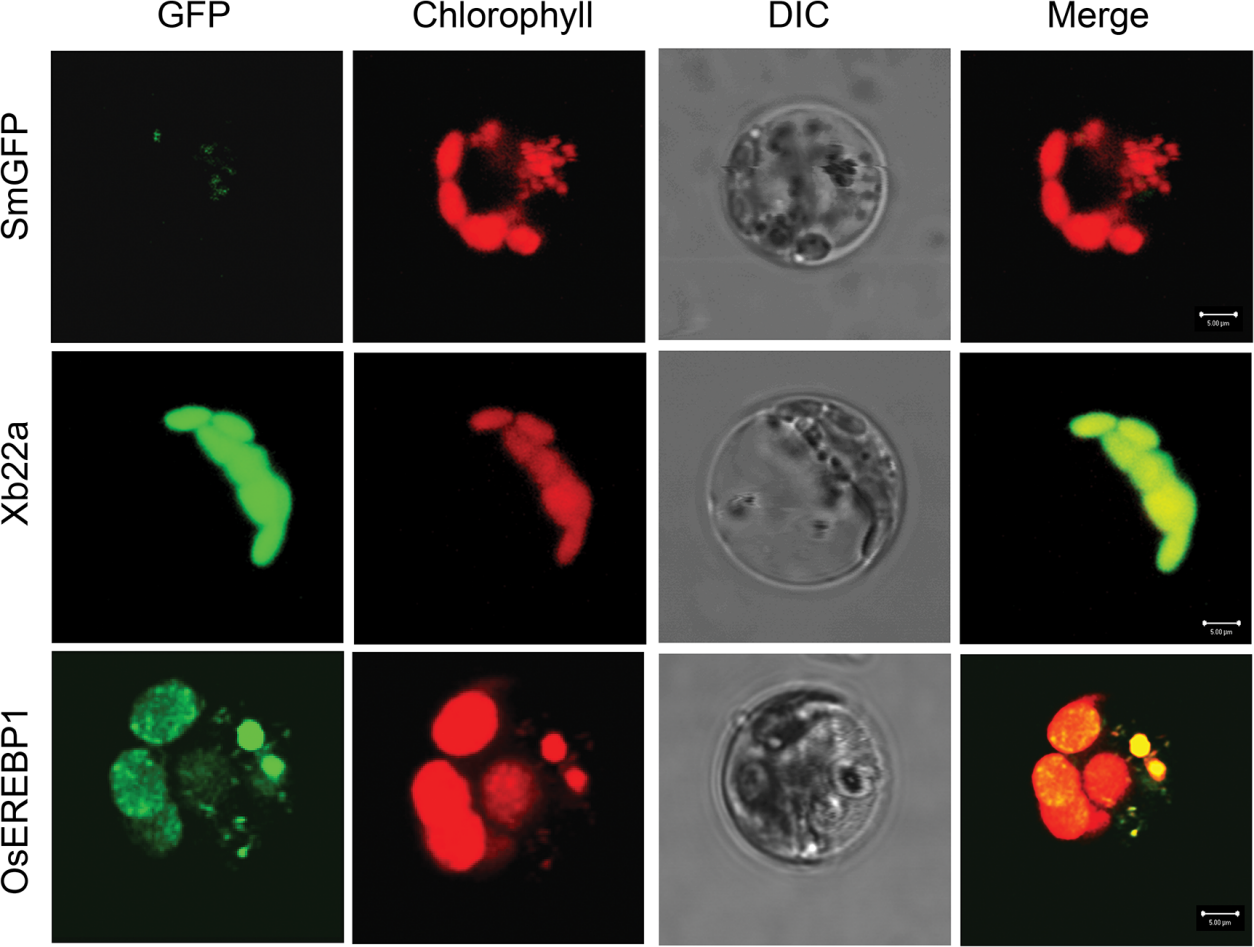
Subcellular localization of OsEREBP1-SmGFP and Xb22a-SmGFP fusion proteins. Confocal micrographs of individual rice protoplast transformed with Ubi:*SmGFP* (SmGFP), Ubi:*Xb22a*-*SmGFP* (Xb22a) or Ubi:*OsEREBP1*-*SmGFP* (OsEREBP1) plasmids. The columns from left are: GFP-flourescence in green (false color), chlorophyll fluorescence in red (false color), differential interference contrast transmission (DIC) and the merged image.

Xb22, identified as an interacting partner of Xa21K668 in yeast two hybrid screens [22], when used as a bait in yeast two-hybrid screen interacted with OsEREBP1. Xb22 has two isoforms of which Xb22a is a protein of 335 amino acids containing PDZ domain, a K-box domain and a tetratricopeptide repeat domain, whereas the second isoform Xb22b is alternatively spliced in the first intron resulting in a protein of 220 amino acids with variable N-terminal region (S1 Fig). The interaction of the two isoforms with OsEREBP1 using the Proquest yeast two-hybrid system (Invitrogen, USA) showed that OSEREBP1 interacts with only Xb22a but not Xb22b (S1 Fig). Several differentially regulated genes in the *OsEREBP1-ox* line that were observed from the microarray data predicted chloroplast/plastid localization. Analysis of the predicted protein sequence of *OsEREBP1* showed a DNA binding domain, a putative nuclear localization signal but no clear chloroplast signal, whereas its interacting protein Xb22a showed presence of a characteristic chloroplast signal (S1 Fig). To investigate the subcellular localization of OsEREBP1 and Xb22a, the pUbi:*Xb22a*-*SmGFP* (Xb22a) or pUbi:*OsEREBP1*-*SmGFP* (OsEREBP1) plasmids were transiently expressed in rice protoplasts by PEG mediated transfections. Confocal microscopy (CLSM) analysis revealed that GFP fluorescence of Xb22a-SmGFP was targeted to chloroplasts and completely overlapped with the red autofluorescence of chlorophyll. The OsEREBP1-SmGFP was localized to chloroplast and GFP fluorescence was seen as distinct punctate patterns in the red background of chlorophyll autofluorescence indicating that the protein is not only chloroplast localized but is associated with chloroplast nucleoids. The control GFP construct alone showed diffused fluorescence (Fig 3).

### *OsEREBP1-ox* plants show increased levels of α-linolenate and phytohormones

Lipids and lipid metabolites act as important regulators of defense responses. Biologically active lipids and precursors of oxylipins are released from plant membranes by the activity of enzymes such as lipases and lipoxygenases [36]. Since six lipases and four chloroplastic LOX genes were significantly up-regulated in the transgenic plants (Table 1), the fatty acid levels were analyzed in the *OsEREBP1-ox* lines in comparison to untransformed control plants in three independent experiments by GC/mass spectrometer and values were calculated as mol% ± SD. Lipids in all the rice leaf samples comprised of mainly four kinds of fatty acids namely 16:0, hexadecanoic acid (palmitic acid), 18:0, octadecanoic acid (stearic acid), 18:2, ∆ 9, 12 octadecadienoic acid (linoleic acid) and 18:3, ∆ 9, 12, 15 octadecatrienoic acid (α-linolenic acid). The molar ratios of these fatty acids in Kitaake was 43.9 ± 3.1, 10.1 ± 1.6, 6.5 ± 1 and 37.8 ± 6.2 respectively, with respect to nonadecanoic acid that was used as internal control. The molar ratios of these fatty acids was 33.4 ± 5.4, 9.1 ± 4, 4.6 ± 1.1 and 51.5 ± 11 in 4–3 and 36.7 ± 3.4, 6.6 ± 0.7, 6.2 ± 0.5 and 49.3 ± 3.3 in 2–4 transgenic lines, respectively (Table 2). The nmoles/fresh weight tissue of 18:3 (octadecatrienoic acid) was 36.4% and 30.4% higher in 4–3 and 2–4 transgenic lines, respectively as compared to Kitaake, whereas the levels of other fatty acids were similar in the transgenic and control plants.

**Table 2:** Fatty acid composition of total lipids extracted from the leaves of 2–3 week old non-transgenic (KIT) and *OsEREBP1-ox* (2–4 and 4–3) plants.

Cultivar	16:0	18:0	18:2	18:3
KIT	43.9±3.1	10.1±1.6	6.5±1	37.8±6.2
4–3	33.4±5.4	9.1±4	4.6±1.1	51.5±11
2–4	36.7±3.4	6.6±0.7	6.2±0.5	49.3±3.3

Values are mol%± S.D (n = 3)

16:0, hexadecanoic acid (palmitic acid);

18:0, octadecanoic acid (stearic acid);

18:2, ∆ 9,12- octadecadienoic acid (linoleic acid);

18:3, ∆ 9,12,15-octadecatrienoic acid (α-linolenic acid)

Several genes related to jasmonate and ABA metabolism were found to be upregulated in *OsEREBP1-ox* plants and so we analyzed the levels of phytohormones in transgenic and non-transformed control plants. Overexpression of *OsEREBP1* induced accumulation of JA and its derivatives collectively known as jasmonates. There was >2-fold increase in the levels of JA, bioactive 7-iso-jasmonyl isoleucine (JA-Ile), OH-JA-Ile and COOH-JA-Ile in the transgenic lines (Table 3), whereas *cis-*12-oxo-phytodienoic acid (*cis-*OPDA) levels were not significantly different. The levels of ABA were ~2-fold increased in the transgenic plants as compared to non-transgenic control whereas salicylic acid (SA) levels which are generally high in rice showed no significant difference. Between the two transgenic lines, considerable variation was observed in 2–4 replicates, which is reflected in the high SD values. However, in spite of this, overall values in 2–4 are still higher as compared to non-transgenic control plants.

**Table 3:** Phytohormone levels in the leaves of OsEREBP1-ox plants v/s Non-transgenic controls.

Phyto-hormones	Kit	2–4	4–3
SA	5617.00 ± 197.2	6118.80 ± 46.3	5533.99 ± 482.23
JA	333.00 ± 40.3	381.50 ± 116.3	759.33 ± 15.05
ABA	26.00 ± 1.4	44.60 ± 6.3	51.89 ± 0.14
JA-Ile	26.00 ± 3.35	51.30 ± 21.99	157.39 ± 3.01
*cis*-OPDA	10.70 ± 1.30	9.80 ± 1.14	13.91 ± 0.39
OH-JA	2.60 ± 2.63	11.90 ± 4.07	37.89 ± 0.90
OH-JA-Ile	0.65 ± 0.02	1.49 ± 0.51	3.77 ± 0.04
COOH-JA-Ile	3.53 ± 0.21	5.94 ± 0.51	6.05 ± 0.03

Values are ng phytohormones g^-1^FW ±S.D (n = 3)

SA, Salicylic acid; JA, Jasmonic acid; ABA, Abscisic acid; *cis*-OPDA, *cis*-12-oxo-phytodienoic acid; JA-Ile, (+)-7-iso-jasmonic acid-L-Ile; COOH-JA-IL.

### *OsEREBP1* expressing transgenic rice shows reduced susceptibility to bacterial blight

**Fig 4:**
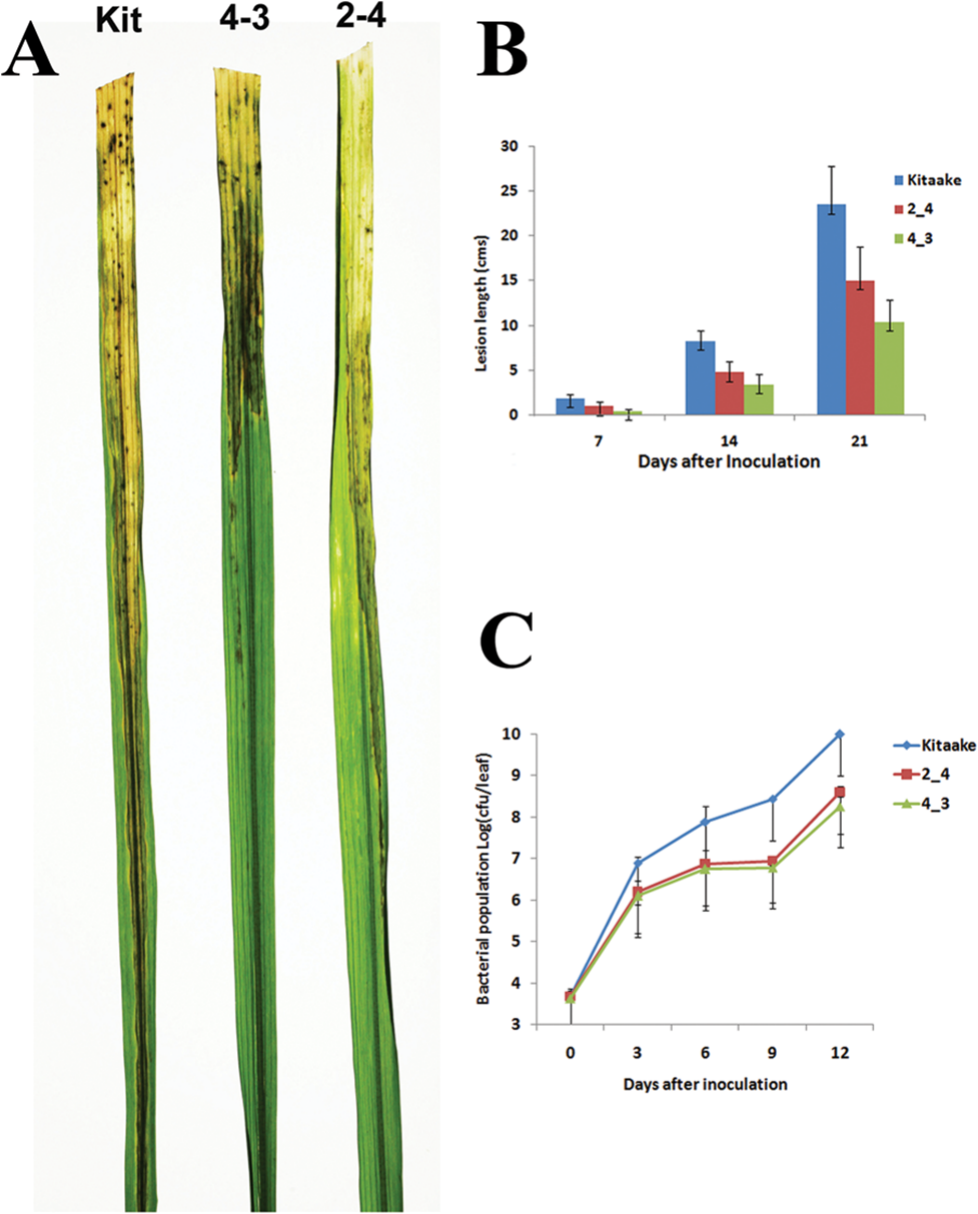
*OsEREBP1-ox* plants show reduced susceptibility to bacterial pathogen Xoo. (A) Leaf lesion development: Six week old plants of Kitaake (control), 4–3 and 2–4 (T3 progeny of transgenic lines) were inoculated by clipping the leaves with a scissor dipped in the Xoo inoculum. Photos of leaves showing lesions 14 days post inoculation. (B) Leaf lesion length: Lesion lengths (in cms) were measured 7, 14 and 21 days post inoculation from 10 inoculated leaves. Values are means ± SD of three replicates. (C) Bacterial growth curves: Three leaves for each of the cultivars 3, 6, 9 and 12 days post inoculation were individually ground in water and plated at various dilutions to get suitable bacterial counts. Each data point represents average and standard deviation of three independent experiments.

*OsEREBP1* was identified as a downstream component of Xa21-mediated resistance to the bacterial pathogen Xoo and hence, we wanted to assess its role in resistance to the pathogen. Xoo is a vascular pathogen and clip inoculations give rise to lesions whose leading edge advances through the mid-vein, causing drying up of the upper regions of the leaf [31]. Six week old transgenic plants over expressing *OsEREBP1* (4–3 and 2–4) and the untransformed control plants (Kit) were inoculated with the BXO43 strain of Xoo. Leaves of Kitaake exhibited typical lesions that extended through the mid-vein along the entire length of the leaf after 14 days of inoculation (DOI), whereas lesions through mid-veins and drying of leaves were distinctly less in the leaves from transgenic lines (Fig 4A). The disease was measured as length of Xoo induced lesions or bacterial population in the inoculated leaves. The transgenic plants showed significant reduction in the lesion lengths (12–15 cm) as compared to the ~22cm in the control after 21 DOI with BXO43 strain (Fig 4B). We quantified the effect of *OsEREBP1* overexpression on bacterial growth by monitoring bacterial population in the leaves of transgenic and control rice plants inoculated with BXO43. After seven DOI, Xoo populations in Kitaake reached approximately 1 X 10^10^ colony forming units per leaf (cfu/leaf) and 1–5 x 10^8^ cfu/leaf in the two transgenic lines (Fig 4C). CFU is a measure of bacterial population within the host and a higher CFU indicates greater susceptibility of the host. A 50–100 fold decrease in the bacterial population was observed in the transgenic lines when compared to the susceptible control Kitaake, indicating a significant difference in the bacterial growth within the plant. These results demonstrate that over expression of *OsEREBP1* attenuates the disease caused by Xoo in rice.

### *OsEREBP1* confers drought and submergence tolerance

**Fig 5:**
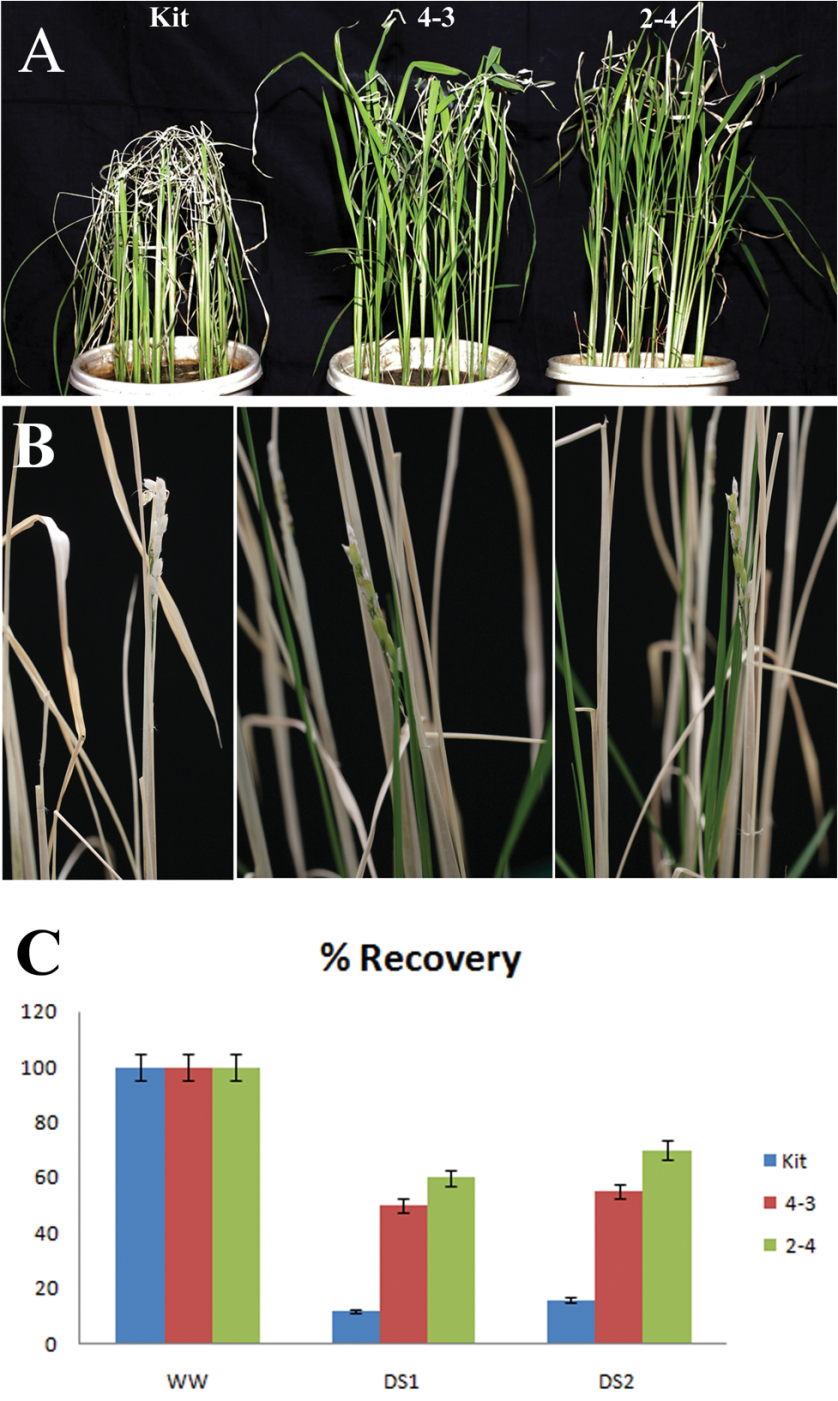
*OsEREBP1-ox* plants show increased tolerance to drought. (A) Photos of transgenic rice plants (4–3 and 2–4) and non-transgenic control (Kit) plants subjected to drought stress for 26 days followed by 7 days of watering. (B) Drought tolerance at the reproductive stage. Drought was imposed by withdrawing water at panicle initiation stage till plants were completely brown before they were re-watered. Recovery was indicated by new tillers which formed panicles and grains following rewatering. (C) Percentage recovery of drought stress plants (DS1 and DS2) compared to well-watered ones (WW). Drought was imposed at four leaf stage (DS1) or panicle initiation or booting stage (DS2) till the plants showed complete browning of leaves. Pots were rewatered and percentage recovery was calculated based on the number of plants that produced new leaves out of the total number of plants per pot. Error bars represent mean of three independent experiments.

Our microarray and qPCR analysis showed that another transcription regulator *OsNAC6* belonging to the NAC family was 1.66-fold up-regulated in the transgenic plants. NAC (NAM, ATAF and CUC) domain containing proteins, another class of plant specific transcription factors form a large family with ~151 predicted members in rice [37] and play important role in biotic as well as abiotic stress responses. Hence, we anticipated that the transgenic plants may endure abiotic stress such as drought. To assess the drought tolerance at the vegetative stage, seedlings of transgenic and non transgenic (NT) control plants grown in a greenhouse were subjected to drought stress at the four leaf stage by withdrawing water for 26 days followed by 1 week of watering. During the course of drought treatment, the transgenic plants showed delay in stress-induced symptoms such as leaf-rolling and wilting as compared to NT plants (Fig 5A). To analyze the drought tolerance at the reproductive stage, plants grown in pots were subjected to continuous water stress at the panicle heading stage till the plants showed complete drying and were subsequently rewatered to allow recovery. The transgenic plants had more number of new tillers, which later formed panicles and grains following rewatering as compared to the control plants (Fig 5B). Transgenic plants over expressing the transcription factor (*OsEREBP1-ox*) also showed better recovery with 50% recovery for 4–3 and 60% for 2–4 as compared to 10% in the non-transgenic control when water stress was given (DS1, Fig 5C). The recovery from drought stress at reproductive stage was 55% for 4–3, 70% for 2–4 and 16% for Kitaake control (DS2, Fig 5C). These results show that over expression of *OsEREBP1* in rice leads to increased drought tolerance at vegetative as well as reproductive stages of growth.

**Fig 6:**
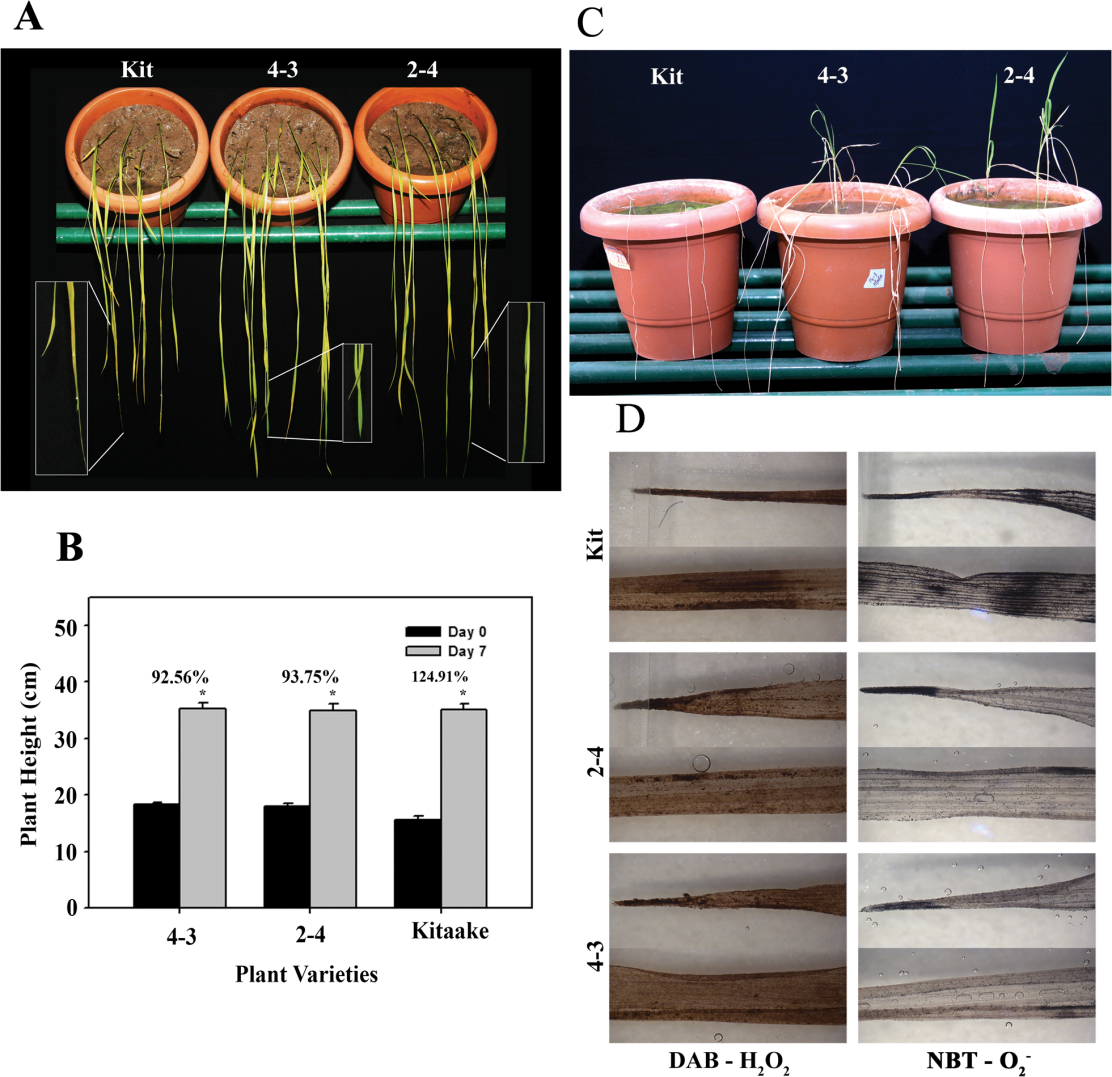
*OsEREBP1* confers submergence tolerance in rice. (A) Two-week old plants were removed from water after 7 days of submergence and photographed. (B) Plant height after submergence treatment. Two-week old plants were submerged for 7 days and plant height of 10 plants was measured at 0 and 7 days after submergence. The error bars represent means ± SD (n = 3) and asterisk indicates that the differences in length were significant (P<0.01) as analyzed by one way ANOVA using SigmaPlot Version 11.0. (C) Plant viability after submergence treatment. Two-week old seedlings of transgenic lines (4–3, 2–4) and Kitaake control were submerged for 14 days and allowed to recover under normal conditions. The plants were scored as viable if they produced new leaves. (D) Accumulation of reactive oxygen species upon submergence. Two-week old seedlings were submerged for 7 days and immediately after desubmergence, the leaves were stained with DAB or NBT to detect hydrogen peroxide or superoxide radicals, respectively.

During anaerobic conditions such as caused by submergence, alcohol fermentation is important for survival of plants. In anaerobic pathway, pyruvate generated by glycolysis is converted to acetaldehyde which is either reduced by alcohol dehydrogenase (ADH) to alcohol or oxidized to acetate by aldehyde dehydrogenase (ALDH). Submergence tolerance in rice is conferred by *Sub1A* which is another AP2/ERF transcription factor, which positively regulates transcription of *Adh1* [14]. Several Adh genes including *Adh1* were upregulated in the *OsEREBP1-ox* plants. In order to assess the role of *OsEREBP1* in submergence tolerance, two week old seedlings of 4–3 and 2–4 (transgenic) and Kitaake (untransformed control) were submerged in water for various time intervals. The rolling and yellowing of leaves following 7 days of submergence was more severe in Kitaake upon desubmergence whereas the upper leaves of 2–4 and 4–3 remained flat and green for a longer period (Fig 6A). Submergence causes leaf and internode elongation and decrease in the rate of plant growth is a measure of tolerance to submergence stress. Plant heights of 14 day old seedlings were measured before and after they were submerged in water for 7 days and the difference in their heights was calculated (Fig 6B). An increase of 125% over the 0 day control in plant height was observed in Kitaake as compared to 92.56% in 4–3 and 93.75% in 2–4 indicating that leaf elongation was greater in Kitaake than in *OsEREBP1-ox* lines after submergence treatment. Similar results were obtained when two week old seedlings were submerged in water for 5 days and their height was compared with plants not subjected to submergence (aerobic conditions). The difference in heights between Kitaake plants subjected to aerobic and submerged conditions was 30% as compared to 12.7% and 11.1% difference in 4–3 and 2–4 respectively (S2 Fig). Upon a more prolonged submergence for 14 days, the withered and dried leaves failed to recover in all the genotypes but there was a significant difference in the emergence and growth of new leaves. More than 80% of plants of 2–4 and ~60% of 4–3 transgenic lines recovered and produced new leaves as compared to 0–28% of Kitaake plants (Fig 6C). These results suggest that constitutive expression of OsEREBP1 increases submergence tolerance and improves recovery from prolonged submerged conditions in rice plants. To assess the injury caused by overproduction of reactive oxygen species (ROS) upon sudden re-exposure to air after submergence, leaves were stained with NBT (nitro-blue tetrazolium) and DAB (3–3’diaminobenzidine tetrahydrochloride) to visualize the accumulation of superoxide O_2_^-^ and H_2_O_2_, respectively immediately following desubmergence. The intensity of blue and brown colors were higher in Kitaake leaves as compared to the transgenic (2–4 and 4–3) leaves (Fig 6D). This suggests that OsEREBP1 enhances submergence recovery and drought tolerance in rice.

## Discussion

Innate immune response in plants involves recognition of pathogen associated molecular patterns (PAMPs) by pathogen recognition receptors (PRRs) in the host. In rice, Xa21 is a PRR which confers broad spectrum resistance against strains of Xoo secreting sulphated peptide Ax21 [17]. Our study show that OsEREBP1 interacts with one of the eight unique Xa21-binding protein (Xb22a) in the Xoo-rice resistance pathway (S1 Fig). *OsEREBP1* belongs to a large family of AP2-domain containing transcription factors and can be classified as group VIIa class of proteins along with 13 members, which are phosphorylated by MAP kinases [4]. *OsEREBP1* is induced in response to the fungal elicitor [38] and is phosphorylated by MAP kinase, OsMPK12 (BWMK1), which upon overexpression in tobacco resulted in expression of PR genes and increased resistance to *Pseudomonas syringae* and *Phytophthora parasitica* infection [39]. In this study, we used the transgenic approach to elucidate the role of *OsEREBP1* in various stress responses. Earlier studies have shown that expression of transcription factors using constitutive promoters causes growth abnormalities and sterility [40–42], whereas constitutive expression of *OsEREBP1* driven by *Ubi* promoter did not cause any undesirable growth phenotypes in our study. Transgenic rice plants constitutively expressing the transcription factor showed attenuation of the disease symptoms upon infection with the bacterial pathogen and increased tolerance to drought and submergence treatment. *OsEREBP1-ox* plants also showed reduced lesions upon infection with virulent strain of *M*. *griseae* (S3 Fig) following increased expression of chloroplastic *Lox* and *PR* genes (Fig 2). Our study reveals that overexpression of transcription factor *OsEREBP1* confers biotic and abiotic stress tolerance in rice and could be a good candidate gene for engineering plants for multiple stress tolerance without any growth defects.

Microarray analysis was performed for expression profiling studies to identify genes regulated by *OsEREBP1* using three biological replicates and all the data has been deposited in public database. However, while analyzing the data hierachical clustering showed that two replicates of control (Kitaake) formed one cluster and two replicates of sample (transgenic line 4-3-1) formed another cluster whereas the third replicate of control and sample did not group with either cluster (S4 Fig). When data from all three replicates was used for analysis *OsEREBP1* did not show upregulation, whereas transcripts levels were shown to be higher in transgenic plants (Fig 1E). Hence, only data from two replicates which showed strong clustering was used for further analysis. We validated the subset of JA and ABA related genes and also *OsEREBP1* using qPCR (Fig 2) and the results confirmed the trend in array data thus obtained. Transcriptome analysis showed high expression of several transcription regulators belonging to the WRKY and NAC family in the transgenic plants. *WRKY* genes included ABA-induced *OsWRKY71* involved in rice defence response [43], Xa-21 binding *OsWRKY62* [44] in addition to *OsWRKY28* and *OsWRKY76* and overexpression of all four genes belonging to WRKYIIa family, conferred resistance to Xoo infection by increased expression of PR10 gene [45]. Among the NAC family of transcription factors, *OsNAC6*, which is induced by ABA and JA and its overexpression confers tolerance to drought and blast infection [42, 46] and two other NAC genes, *ONAC122* and *ONAC131*, which are JA-responsive genes conferring resistance to blast infection [47] were upregulated in the *OsEREBP1-ox* lines. Immune response is specifically induced in cultured rice cells by another PAMP, flagellin from incompatible strains of bacterial pathogen *Acidovorax avenae* and *OsEREBP1* and *OsNAC6* were among the genes that were induced by inoculation with incompatible as well as flagellin-deficient strains of *A*.*avenae* [48].

Overexpression of *OsEREBP1* results in increased levels of linolenic acid, jasmonates and ABA and corresponding increased expression of the key genes, *LOX* and *NCED* involved in the biosynthesis of jasmonic acid and ABA, respectively, in transgenic plants. The initial stage of JA biosynthesis occurs in plastids and involves the conversion of α-linolenic acid by chloroplastic LOX to OPDA, which is transported to peroxisomes for conversion to JA. The bio-active form of jasmonic acid is (3R, 7S)-*N*-jasmonoyl-L-isoleucine (JA-Ile). It is generated in the cytosol by the conjugation of JA with isoleucine [49]. JA-Ile enters the nucleus, binds to COI1 receptor and activates JA responsive genes. The levels of JA-Ile are controlled by catabolism and deactivation of the hormone. The ω-oxidation pathway catalyzed by the CYP94B3 and CYP93C1, in which JA-Ile is converted to12-hydroxy-JA-Ile (12-OH-JA-Ile) and then further oxidized to dicarboxy-JA-Ile (12-COOH-JA-Ile), is a major route for catabolism of the hormone [50]. Similarly, ABA biosynthesis involves oxidative cleavage of cis-isomers of violaxanthin and neoxanthin by NCED in the plastids to produce the cytoplasmic precursor xanthoxin before final conversion to ABA [51]. Transcription factors may be imported into chloroplast to regulate transcription of chloroplast genes. *In silico* analysis of *Arabidopsis* genes encoding putative transcription factors indicate that ~100 TFs are likely to be imported into the chloroplasts which include genes with AP2-, LOB- Zn finger- and TPR- domain containing proteins [52]. Among the several proteins in the proteome analysis of a highly enriched fraction of transcriptionally active chromosomes from chloroplasts of *Spinacia oleracea* that were identified in structuring of plastid nucleoid core included a member of AP2-EREBP family of transcription factor [53]. Although, OsEREBP1 does not show presence of chloroplast localization signal, it interacts with Xb22a isoform, which shows the presence of a strong chloroplast transit peptide and is localized to chloroplast (Fig 3). We found that OsEREBP1 protein is localized to chloroplast nucleoids where it probably up-regulates the expression of the chloroplastic *LOX* and *NCED* thereby triggering the JA and ABA biosynthetic pathways.

ABA biosynthesis is induced during abiotic stress conditions such as drought and serves as chemical signal leading to adaptation to stress conditions. The *OsEREBP1-ox* plants showed increased expression of ABA-related genes such as *OsNCED1*, *AP39*, *LEA5* and *CatB*. Overexpression of *AP39* leads to upregulation of *NCED1*and increase in endogenous levels of ABA [35] and the ABA induced during water stress prevents H_2_O_2_ accumulation by upregulating *CatB* expression in rice [51]. Accumulation of H_2_O_2_ was reduced in *OsEREBP1-ox* leaves during drought-like situation induced by desubmergence with increased expression of *Adh1*, similar to the increased ABA-responsiveness observed by overexpression of *Sub1A* in rice [54]. Thus, we surmise that *OsEREBP1* confers tolerance to stresses caused during submergence and dehydration during desubmergence by regulating the ABA levels in rice.

Jasmonic acid and its derivatives are fatty acid based oxylipins involved in complex network of jasmonate signaling and hormone cross talk in stress responses and development [55]. Pathogen attack or wounding, increases lipase activity in plants, with the generation of unsaturated fatty acids such as octadecatrienoic acids, which showed elevated levels in the *OsEREBP1-ox* plants and form the substrate for LOX triggering synthesis of oxylipins such as jasmonates. Four *LOX* genes found in *A*. *thaliana* contribute to JA synthesis in response to wounding with *LOX6* being involved in long distance signaling [56]. Suppression of *LOX2* resulted in absence of JA accumulation in *A*. *thaliana* leaves, while overexpression of *TomLOX2* led to elevated JA-biosynthesis in tomato indicating that chloroplastic *LOX* is involved in wound-induced JA synthesis [57, 58]. Homeostasis of jasmonates involving catabolic turnover of jasmonate derivatives is suggested by increased expression of a wound-inducible *CYP94C1*, which catalyzes the conversion of JA-Ile to 12COOH-JA-Ile [59]. *OsEREBP1-ox* plants accumulated higher levels of endogenous jasmonic acid, bioactive JA-Ile, 12OH-JA-Ile and 12COOH-JA-Ile and showed higher expression of the helix-loop-helix transcription regulator *RERJ1*, linalool synthase, jasmonate-induced protein and other PR genes. JA accumulation has been shown to tightly regulate expression of *RERJ1*, with localized expression in response to wound stress and in entire leaf during drought [60, 61]. Previous studies show that pathogenic attack increased endogenous levels of JA and application of JA to plants activated expression of PR genes and induced blast resistance in rice [62]. JA has been shown to have a significant role in rice resistance to Xoo [63] with JA application on rice plants inducing expression of *OsLiS* (Linalool synthase) resulting in accumulation of volatile monoterpene linalool, which induces resistance to Xoo infection [64]. Our gene expression study suggests that activation of jasmonate signaling pathway results in resistance to bacterial and fungal pathogens by the monoterpene linalool and drought tolerance by activation of PR genes mediated by JA-induced *RERJ1* transcription regulator. JA and ABA were synergistic for the expression of helix-loop-helix *OsHLH148* and its interacting partner *OsJAZ1* in rice plants exposed to drought stress, with the overexpression of *OsHLH148* leading to upregulation of several AP2/ERF genes including *AP59* [65]. A cross-talk between JA and ABA signaling pathways in conferring drought tolerance is evident with the *OsEREBP1-ox* plants showing increased transcript levels of *OsbHLH148*, *OsJAZ1* and *AP59* genes. ABA is one of the phytohormones that also regulate seed dormancy and its concentration in a specific tissue is determined by the process of biosynthesis, catabolism, compartmentation and transport. Among the members of ABA biosynthesis enzymes, NCED is the foremost one which catalyzes the regulating step of this pathway and of the five *OsNCED* gene family members present in rice, gene expression analysis reveals that the transcript level of *OsNCED2* gene is abundant in seed [66] while *OsNCED1* is mainly expressed in rice leaves [67] and *OsNCED3* may play a role in controlling dormancy and germination [68]. We did not observe any difference in the germination of seeds between the transgenic and control lines and microarray results showed that only *OsNCED1* is upregulated in the transgenic plants. Moreover, the levels of ABA were analyzed only in the leaf tissue and not in the seeds.

**Fig 7:**
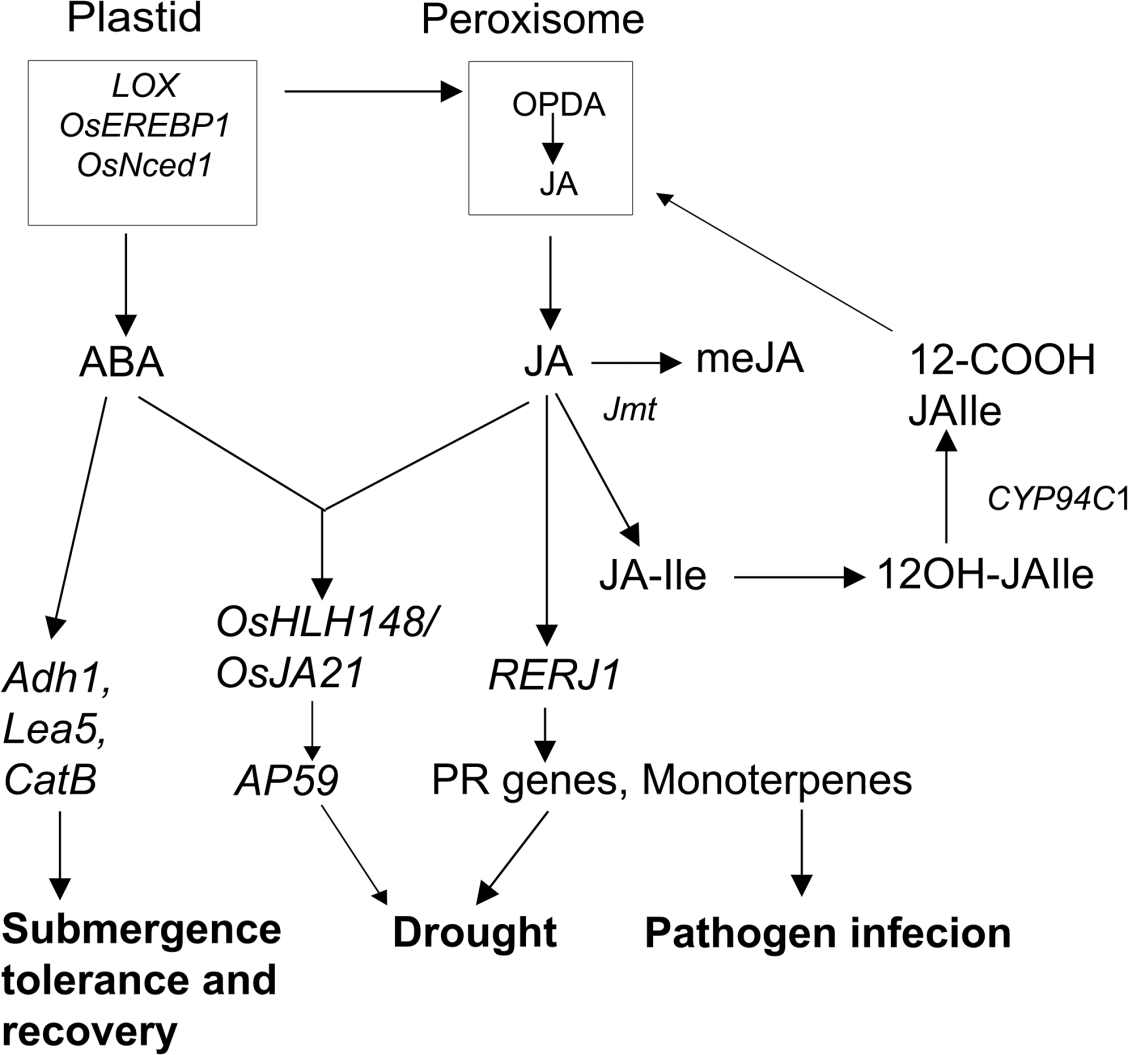
Model for role of *OsEREBP1* in biotic and abiotic stress responses. Increased expression of *OsEREBP1* results in accumulation of jasmonates and ABA and induction of specific subsets of defense genes leading to biotic and abiotic stress tolerance. JA regulates transcription factor *RERJ1* and induces expression of JA-responsive genes resulting in increased tolerance to pathogen infection and drought stress. A cross talk between ABA and JA signalling mediated by OsHLH148/OsJaz1 interaction results in upregulation of transcripts encoding ERF’s such as *AP59* associated with drought acclimation. ABA independently enhances recovery from submergence and provides protection against drought during desubmergence by inducing expression of *Lea5* and *CatB* genes.

Based on the transcriptome analysis of *OsEREBP1-ox* plants and the phenotypes observed, we propose a model for the possible mechanism for OsEREBP1 mediated responses (Fig 7). Increased expression of *OsEREBP1* in response to various biotic signals or its overexpression leads to accumulation of jasmonates and ABA and induction of specific subsets of defense genes ultimately resulting in abiotic and biotic stress tolerance in rice. JA confers resistance to bacterial and fungal pathogens and drought stress by regulating the transcription factor *RERJ1* which activates its target PR genes. JA and ABA also synergistically regulate another transcription factor *OsHLH148* thereby conferring drought tolerance. ABA induces the expression of genes related to oxidative stress that occurs during prolonged submergence in water and dehydration during subsequent desubmergence. Promoter analysis of the differentially regulated genes for transcription factor specific binding motifs would identify the direct targets of the *OsEREBP1* and the various transcriptional regulators in the individual signaling pathways.

## Supporting Information

S1 Fig OsEREBP1 interacts with Xb22a isoform. X-gal ‘on-plate’ detection for interactions of Xb22a and Xb22b isoforms with OsEREBP1 (AP2). Xb22a and Xb22b baits were expressed in pDEST32 (pDEST32/Xb22a and pDEST32/Xb22b, respectively) and OsEREBP1 prey was expressed in pDEST22 (pDEST22/AP2). Yeast strain MaV203 harboured the following combination of plasmids: 1a-4a) Four transformants of pDEST32/Xb22a + pDEST22/AP2; 1b-4b) Four transformants of pDEST32/Xb22b + pDEST22/AP2; 5) pDEST32 + pDEST22; 6) Positive control vectors pEXP32/Krev1 + pEXP22/RalGDS-wt; 7) pEXP32/Krev1 + pEXP22/RalGDS-m1; 8) pEXP32/Krev1 + pEXP22/RalGDS-m2; 9a) pDEST32/Xb22a + pDEST22; 9b) pDEST32/Xb22b + pDEST22; 10) pDEST32 + pDEST22/AP2. The Xb22a interacted strongly with OsEREBP1 as indicated by blue color in β-galactosidase assays for *lacZ* reporter gene (1a-4a). However, no color developed when Xb22b/pDEST32 was interacted with AP2/pDEST22 indicating that Xb22b does not interact with OsEREBP1 (1b-4b). The empty vector pDEST22 did not interact with Xb22a/pDEST32, Xb22b/pDEST32 and pDEST32 (9a, 9b) did not interact with and AP2/pDEST22 (10), whereas positive control (supplied with the kit) showed blue color (6,7,8).
(TIF)

S2 Fig *OsEREBP1* confers submergence tolerance in rice. Two-week old plants were submerged for 5 days and the height of the plants was measured following submergence treatment. Plants grown under aerobic conditions were used as control. The error bars represent standard deviation of readings from 10 plants and asterisk indicates that the differences in length were significant (P<0.01).
(TIF)

S3 Fig *OsEREBP1-ox* plants show resistance to blast fungus. The latest fully expanded leaves from 3-week old control (Kit) and transgenic plants (2–4 and 4–3) were spotted with conidial spores (4x 10^4^ conidia/ml) suspended in 0.25% gelatin. The leaves were incubated in growth chamber on moist filter paper with 16h/8h of light/dark regime and were observed for lesion development.
(TIF)

S4 Fig Hierarchical clustering of microarray data. Of the three replicates of control (Kitaki) vs. sample (4-3-1), rep_3 and rep_4 of kitaki form one cluster, rep_3 and rep_4 of 4-3-1 form another cluster, whereas rep_5 of Kitaki and 4-3-1 are distinct.
(TIF)

S1 Table List of primers used in this study. (DOC)

S2 Table List of genes expressed differentially in *OsEREBP1* transgenic rice leaves. (XLS)

## References

[1] Gao G, Zhong Y, Guo A, Zhu Q, Tang W, Zheng W, et al (2006) DRTF: a database of rice transcription factors. Bioinformatics 22: 1286‒1287. 16551659 10.1093/bioinformatics/btl107

[2] Todaka D, Nakashima K, Shinozaki K, Shinozaki KY (2012) Toward understanding transcriptional regulatory networks in abiotic stress responses and tolerance in rice. Rice 5: 6 10.1186/1939-8433-5-6 24764506 PMC3834508

[3] Khong GN, Richaud F, Coudert Y, Pati PK, Santi C, Perin C, et al (2008) Modulating rice stress tolerance by transcription factors. Biotechnology and Genetic Engineering Rev 25: 381‒404. 21412363 10.5661/bger-25-381

[4] Nakano T, Suzuki K, Fujimura T, Shinshi H (2006) Genome-wide Analysis of the ERF gene family in Arabidopsis and Rice. Plant Physiol 140: 411‒432. 16407444 10.1104/pp.105.073783 PMC1361313

[5] Sharoni AM, Nuruzzaman M, Satoh K, Shimizu T, Kondoh H, Sasaya T, et al (2011) Gene structures, classification and expression models of the AP2/EREBP transcription factor family in rice. Plant Cell Physiol 52: 344–360. 10.1093/pcp/pcq196 21169347

[6] Park JM, Park CJ, Lee SB, Ham BK, Shin R, Paek KH (2001) Overexpression of the tobacco *Tsi1* gene encoding an EREBP/AP2–type transcription factor enhances resistance against pathogen attack and osmotic stress in tobacco. The Plant Cell 13: 1035–1046. 11340180 10.1105/tpc.13.5.1035 PMC135557

[7] Dubouzet JG, Sakuma Y, Ito Y, Kasuga M, Dubouzet EG, Miura S, et al (2003) *OsDREB* genes in rice, *Oryza sativa* L., encode transcription activators that function in drought-, high-salt- and cold-responsive gene expression. Plant Journal. 33, 751‒763. 12609047 10.1046/j.1365-313x.2003.01661.x

[8] Kasuga M, Miura S, Shinozaki K, Shinozaki KY (2004) A combination of the *Arabidopsis* DREB1A gene and stress-inducible *RD29A* promoter improved drought- and low-temperature stress tolerance in tobacco by gene transfer. Plant Cell Physiology. 45: 346‒350. 15047884 10.1093/pcp/pch037

[9] Sakuma Y, Maruyama K, Osakabe Y, Qin F, Seki M, Shinozaki K, et al (2006) Functional analysis of an *Arabidopsis* transcription factor, DREB2A, involved in drought-responsive gene expression. The Plant Cell 18: 1292–1309. 16617101 10.1105/tpc.105.035881 PMC1456870

[10] Cao Y, Song F, Goodmand RM, Zheng Z (2006) Molecular characterization of four rice genes encoding ethylene-responsive transcriptional factors and their expressions in response to biotic and abiotic stress. J of Plant Physiol 163: 1167‒1178. 16436304 10.1016/j.jplph.2005.11.004

[11] Lin R, Zhao W, Meng X, Peng YL (2007) Molecular cloning and characterization of a rice gene encoding AP2/EREBP-type transcription factor and its expression in response to infection with blast fungus and abiotic stresses. Physiol and Mol Plant Pathol 70: 60–68.

[12] Karaba A, Dixit S, Greco R, Aharoni A, Trijatmiko KR, Marsch-Martinez N, et al (2007) Improvement of water use efficiency in rice by expression of HARDY, an Arabidopsis drought and salt tolerance gene. Proc Natl Acad Sci U S A 104: 15270–15275. 17881564 10.1073/pnas.0707294104 PMC1986572

[13] Oh SJ, Kim YS, Kwon CW, Park HY, Jeong JS, Kim JK (2009) Overexpression of the transcription factor AP37 in rice improves grain yield under drought conditions. Plant Physiol 150: 1368–1379. 10.1104/pp.109.137554 19429605 PMC2705040

[14] Xu K, Xu X, Fukao T, Canlas P, Rodriguez MR, Heuer S, et al (2006) Sub1A is an ethylene-response-factor-like gene that confers submergence tolerance to rice. Nature 442: 705–708. 16900200 10.1038/nature04920

[15] Quan R, Hu S, Zhang Z, Zhang H, Zhang Z, Huang R (2010) Overexpression of an ERF transcription factor TSRF1 improves rice drought tolerance. Plant Biotech J 8: 476–488. 10.1111/j.1467-7652.2009.00492.x 20233336

[16] Datta K, Baisakh N, Ganguly M, Krishnan S, Shinozaki KY, Datta SK (2012) Overexpression of Arabidopsis and Rice stress genes’ inducible transcription factor confers drought and salinity tolerance to rice. Plant Biotech J 10: 579‒586. 10.1111/j.1467-7652.2012.00688.x 22385556

[17] Lee SW, Han SW, Bartley LE, Ronald PC (2006) Unique characteristics of Xanthomonas oryzae pv. oryzae AvrXa21 and implications for plant innate immunity. Proc Natl Acad Sci U S A 103: 18395–18400. 17082309 10.1073/pnas.0605508103 PMC1693675

[18] Lee SW, Han SW, Sririyanum M, Park CJ, Seo YS, Ronald PC (2009).A Type I–secreted, sulfated peptide triggers XA21-mediated innate immunity. Science 326: 850‒853. 10.1126/science.1173438 19892983

[19] Song WY, Wang GL, Chen LL, Kim HS, Pi LY, Holsten T, et al (1995) A receptor kinase-like protein encoded by the rice disease resistance gene, Xa21. Science 270: 1804‒1806. 8525370 10.1126/science.270.5243.1804

[20] Liu GZ, Pi LY, Walker JC, Ronald PC, Song WY (2002) Biochemical characterization of the kinase domain of the rice disease resistance receptor-like kinase XA21. J Biol Chem 277: 20264–20269. 11927577 10.1074/jbc.M110999200

[21] Park CJ, Peng Y, Chen X, Dardick C, Ruan D, et al (2008) Rice XB15, a protein phosphatase 2C, negatively regulates cell death and XA21-mediated innate immunity. PLoS Biol 6, e231 doi: 10.1371/journal.pbio. 0060231 18817453 10.1371/journal.pbio.0060231 PMC2553837

[22] Seo YS, Chern M, Bartley LE, Han M, Jung KH, Lee I, et al (2011) Towards establishment of a rice stress response Interactome. PLoS Genetics 7: e1002020 10.1371/journal.pgen.1002020 21533176 PMC3077385

[23] Park CJ, Bart R, Chern M, Canlas PE, Bai W, Ronald PC (2010) Overexpression of the endoplasmic reticulum chaperone BiP3 regulates XA21-mediated innate immunity in rice. PLoS ONE 5(2): e9262 10.1371/journal.pone.0009262 20174657 PMC2822859

[24] Chern M, Fitzgerald HA, Canlas PE, Navarre DA, Ronald PC (2005) Overexpression of a rice NPR1 homolog leads to constitutive activation of defense response and hypersensitivity to light. Mol Plant Microbe Interactions 18: 511‒520. 15986920 10.1094/MPMI-18-0511

[25] Han MJ, Jung KH, Yi G, Lee DY, An G (2006) Rice immature pollen 1 (RIP1) is a regulator of late pollen development. Plant Cell Physiol 47: 1457‒1472. 16990291 10.1093/pcp/pcl013

[26] Bart R, Chern M, Park C-J, Bartley L, Ronald PC (2006) A novel system for gene silencing using siRNAs in rice leaf and stem-derived protoplasts. Plant Methods 2:13 16808845 10.1186/1746-4811-2-13 PMC1524957

[27] Zhang Y, Su J, Duan S, Ao Y, Dai J, Liu J, et al (2011) A highly efficient rice green tissue protoplast system for transient gene expression and studying light/chloroplast-related processes. Plant Methods 7:30 10.1186/1746-4811-7-30 21961694 PMC3203094

[28] Griffiths G, Leverentz M, Silkowski H, Gill N, Sanchez-Serrano JJ (2000) Lipid hydroperoxide levels in plant tissues. J Exp Bot 51: 1363‒1370. 10944149

[29] Vadassery J, Reichelt M, Hause B, Gershenzon J, Boland W, Mithöfer A (2012) CML42-mediated calcium signaling coordinates responses to Spodoptera herbivory and abiotic stresses in Arabidopsis. Plant Physiol 159: 1159–1175. 10.1104/pp.112.198150 22570470 PMC3387702

[30] Fonseca S, Chini A, Hamberg M, Adie B, Porzel R, Kramell R, et al (2009) (+)-7-Iso-jasmonoyl-L-isoleucine is the endogenous bioactive jasmonate. Nature Chem Biol 5: 344–350. 10.1038/nchembio.161 19349968

[31] Kauffman HE, Reddy APK, Hsieh SPV, Marca SD (1973) An improved technique for evaluation of resistance of rice varieties to *Xanthomonas oryzae* . Plant Disease Reporter 57: 537‒541.

[32] Vadez V, Rao S, Kholova J, Krishnamurthy L, Kashiwagi J, Ratnakumar P, et al (2008) Roots research for legume tolerance to drought: Quo vadis? Journal of Food Legumes 21: 77‒85.

[33] Fukao T, Xu K, Ronald PC, Bailey-Serres J (2006) A variable cluster of ethylene response factor-like genes regulates metabolic and developmental acclimation responses to submergence in rice. The Plant Cell 18: 2021‒2034. 16816135 10.1105/tpc.106.043000 PMC1533987

[34] Fitzgerald HA, Chern MS, Navarre R, Ronald PC (2004) Overexpression of (*At*) NPR1 in Rice Leads to a BTH-and Environment-Induced Lesion-Mimic/Cell Death phenotype. Mol Plant Microbe Interactions 17: 140‒151. 14964528 10.1094/MPMI.2004.17.2.140

[35] Yaish MW, El-kereamy A, Zhu T, Beatty PH, Good AG, Bi YM, et al (2010) The APETALA-2-like transcription factor OsAP2-39 controls key interactions between abscisic acid and gibberellin in rice. PLoS Genet 6: e1001098 10.1371/journal.pgen.1001098 20838584 PMC2936520

[36] Shah J (2005) Lipids, lipases, and lipid-modifying enzymes in plant disease resistance. Ann Rev Phytopathol 43: 229–60. 16078884 10.1146/annurev.phyto.43.040204.135951

[37] Nuruzzaman M, Manimekalai R, Sharoni AM, Satoh K, Kondoh H, Ooka H, et al (2010) Genome-wide analysis of NAC transcription factor family in rice. Gene 465: 30–44. 10.1016/j.gene.2010.06.008 20600702

[38] Kim CY, Lee S, Park HC, Chang CG, Cheong YH, Choi YJ, et al (2000) Identification of rice blast fungal elicitor-responsive genes by differential display analysis. Mol Plant Microbe Interactions 13: 470‒474. 10755311 10.1094/MPMI.2000.13.4.470

[39] Cheong YH, Moon BM, Kim JK, Kim CY, Kim MC, Kim IH, et al (2003) BWMK1, a rice Mitogen-Activated Protein Kinase, locates in the nucleus and mediates pathogenesis-related gene expression by activation of a transcription factor. Plant Physiol 132: 1961‒1972. 12913152 10.1104/pp.103.023176 PMC181281

[40] Ito Y, Katsura K, Maruyama K, Taji T, Kobayashi M, Seki M, et al (2006) Functional analysis of rice DREB1/CBF-type transcription factors involved in cold-responsive gene expression in transgenic rice. Plant Cell Physiol 47: 141–153. 16284406 10.1093/pcp/pci230

[41] Hu H, You J, Fang Y, Zhu X, Qi Z, Xiong L (2008) Characterization of transcription factor gene SNAC2 conferring cold and salt tolerance in rice. Plant Mol Biol 67: 169‒181. 10.1007/s11103-008-9309-5 18273684

[42] Nakashima K, Tran LS, Van Nguyen D, Fujita M, Maruyama K, Todaka D, et al (2007) Functional analysis of a NAC-type transcription factor OsNAC6 involved in abiotic and biotic stress responsive gene expression in rice. Plant J 51: 617–630. 17587305 10.1111/j.1365-313X.2007.03168.x

[43] Liu X, Bai X, Wang X, Chu C (2007) OsWRKY71, a rice transcription factor, is involved in rice defense response. J Plant Physiol 164: 969‒979. 16919842 10.1016/j.jplph.2006.07.006

[44] Peng Y, Bartley LE, Chen X, Dardick C, Chern M, Ruan R, et al (2008) OsWRKY62 is a negative regulator of basal and Xa21-mediated defense against Xanthomonas oryzae pv. oryzae in rice. Mol Plant 1: 446–458. 10.1093/mp/ssn024 19825552

[45] Peng Y, Bartley LE, Canlas P, Ronald PC (2010) OsWRKY IIa transcription factors modulate rice innate immunity. Rice 3: 36‒42. 21961049 10.1007/s12284-010-9039-6 PMC3175632

[46] Ohnishi T, Sugahara S, Yamada T, Kikuchi K, Yoshiba Y, Hirano HY, et al (2006) *OsNAC6*, a member of the NAC family, is induced by various stresses in rice. Genes Genet. Sys 80: 135‒139. 10.1266/ggs.80.135 16172526

[47] Sun L, Zhang H, Li D, Huang L, Hong Y, Ding XS, et al (2013) Functions of rice NAC transcriptional factors, ONAC122 and ONAC131, in defense responses against *Magnaporthe grisea* . Plant Mol Biol 81: 41‒56. 10.1007/s11103-012-9981-3 23103994

[48] Fujiwara S, Tanaka N, Kaneda T, Takayama S, Isogai A, Che FS (2004) Rice cDNA microarray-based gene expression profiling of the response to flagellin perception in cultured rice cells. Mol Plant Microbe Interactions 17: 986‒998. 15384489 10.1094/MPMI.2004.17.9.986

[49] Staswick P.E., and Tiryaki I. (2004). The oxylipin signal jasmonic acid is activated by an enzyme that conjugates it to isoleucine in *Arabidopsis* . Plant Cell. 16, 2117–2127. 15258265 10.1105/tpc.104.023549 PMC519202

[50] Koo AJK, and Howe GA (2012) Catabolism and deactivation of the lipid derived hormone jasmonoyl-isoleucine. Frontiers in Plant Sci 3: 10.3389/fpls.2012.00019 PMC3355578 22639640

[51] Ye N, Jia L, Zhang J (2012) ABA signal in rice under stress conditions. Rice 5: 1 10.1186/1939-8433-5-1 24764501 PMC3834477

[52] Wagner R, Pfannschmidt T (2006) Eukaryotic transcription factors in plastids—Bioinformatic assessment and implications for the evolution of gene expression machineries in plants. Gene 381: 62‒70. 16934950 10.1016/j.gene.2006.06.022

[53] Melonek J, Matros A, Trösch M, Mock HP, Krupinska K (2012) The core of chloroplast nucleoids contains architectural SWIB domain proteins. The Plant Cell 24: 3060–3073. 10.1105/tpc.112.099721 22797472 PMC3426132

[54] Fukao T, Yeung E, Bailey-Serres J (2011) The submergence tolerance regulator SUB1A mediates crosstalk between submergence and drought tolerance in rice. The Plant Cell 23: 412‒427. 10.1105/tpc.110.080325 21239643 PMC3051255

[55] Wasternack C, Hause B (2013) Jasmonates: biosynthesis, perception, signal transduction and action in plant stress response, growth and development. An update to the 2007 review in Annals of Botany. Ann Bot 111: 1021‒58. 10.1093/aob/mct067 23558912 PMC3662512

[56] Chauvin A, Caldelari D, Wolfender JC, Farmer EE (2013) Four 13-lipoxygenases contribute to rapid jasmonate synthesis in wounded Arabidopsis thaliana leaves: a role for lipoxygenase 6 in responses to long-distance wound signals. New Phytologist 197: 566‒575. 10.1111/nph.12029 23171345

[57] Bell E, Creelman RA, Mullet JE (1995) A chloroplast lipoxygenase is required for wound-induced jasmonic acid accumulation in *Arabidopsis* . Proc Natl Acad Sci U S A 92: 8675‒8679. 7567995 10.1073/pnas.92.19.8675 PMC41029

[58] Yan L, Zhai Q, Wei J, Li S, Wang B, Huang T, et al (2013) Role of tomato Lipoxygenase D in wound-induced Jasmonate biosynthesis and plant immunity to insect herbivores. PLoS Genet 9: e1003964. doi:10.1371. 10.1371/journal.pgen.1003964 24348260 PMC3861047

[59] Heitz T, Widemann E, Lugan R, Miesch L, Ullmann P, Désaubry L (2012) Cytochromes P450 CYP94C1 and CYP94B3 catalyze two successive oxidation steps of plant hormone jasmonoyl-isoleucine for catabolic turnover. J Biol Chem 287: 6296‒6306. 10.1074/jbc.M111.316364 22215670 PMC3307330

[60] Kiribuchi K, Jikumaru Y, Kaku H, Minami E, Hasegawa M, Kodama O, et al (2005) Involvement of the basic helix–loop–helix transcription factor RERJ1 in wounding and drought stress responses in rice plants. Biosci Biotech Biochem 69: 1042‒1044. 15914931 10.1271/bbb.69.1042

[61] Miyamoto K, Shimizu T, Mochizuki S, Nishizawa Y, Minami E, Nojiri H, et al (2013) Stress induced expression of the transcription factor RERJ1 is tightly regulated in response to jasmonic acid accumulation in rice. Protoplasma 250: 241‒249. 10.1007/s00709-012-0400-z 22456953

[62] Mei C, Qi M, Sheng G, Yang Y (2006) Inducible overexpression of a rice allene oxide synthase gene increases the endogenous jasmonic acid level, PR gene expression, and host resistance to fungal infection. Mol Plant Microbe Interactions 19: 1127–1137. 17022177 10.1094/MPMI-19-1127

[63] Yamada S, Kano A, Tamaoki D, Miyamoto A, Shishido H, Miyoshi S, et al (2012) Involvement of OsJAZ8 in jasmonate-induced resistance to bacterial blight in rice. Plant Cell Physiol 53: 2060–2072. 10.1093/pcp/pcs145 23104764

[64] Taniguchi S, Shinonaga YH, Tamaoki D, Yamada S, Akimitsu K, Gomi K (2014) Jasmonate induction of the monoterpene linalool confers resistance to rice bacterial blight and its biosynthesis is regulated by JAZ protein in rice. Plant Cell and Environ 37: 451–461. 10.1111/pce.12169 23889289

[65] Seo JS, Joo J, Kim MJ, Kim YK, Nahm BH, Song SI, et al (2011) OsbHLH148, a basic helix-loop-helix protein, interacts with OsJAZ proteins in a jasmonate signaling pathway leading to drought tolerance in rice. The Plant J 65: 907–921. 10.1111/j.1365-313X.2010.04477.x 21332845

[66] Zhu G, Ye N, Zhang J (2009) Glucose-induced delay of seed germination in rice is mediated by the suppression of ABA catabolism rather than an enhancement of ABA biosynthesis. Plant Cell Physiol 50: 644–651. 10.1093/pcp/pcp022 19208695

[67] Ye N, Zhu G, Liu Y, Li Y, Zhang J (2011) ABA Controls H2O2 Accumulation Through the Induction of OsCATB in Rice Leaves Under Water Stress. Plant Cell Physiol 52: 689–698. 10.1093/pcp/pcr028 21398647

[68] Liu F, Zhang H, Wu G, Sun J, Hao L, Ge X, Yu J, Wang W (2011) Sequence variation and expression analysis of seed dormancy- and germination-associated ABA- and GA-related genes in rice cultivars. Frontiers in Plant Science 10.3389/fpls.2011.00017 PMC3355514 22629259

