# Supplementary figures and images for: Overexpression of an AP2/ERF Type Transcription Factor OsEREBP1 Confers Biotic and Abiotic Stress Tolerance in Rice
Source: PLoS One. 2015 Jun 2;10(6):e0127831. doi: 10.1371/journal.pone.0127831 (PMC4452794; doi:10.1371/journal.pone.0127831)

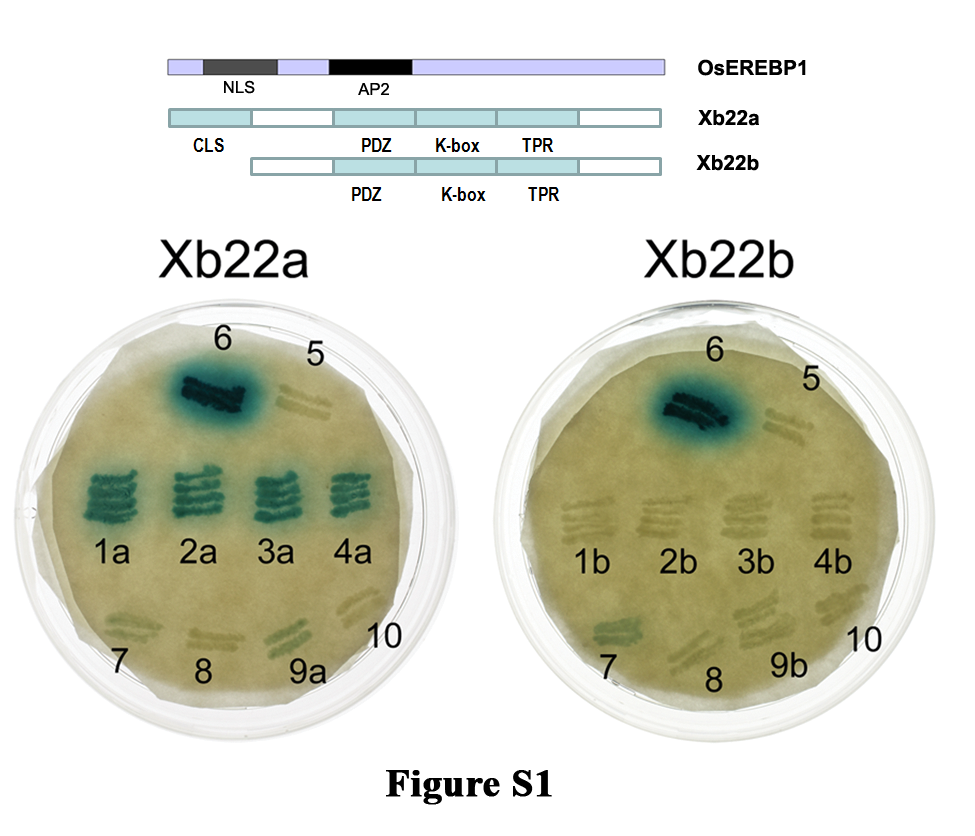

Supplement: S1 Fig — X-gal ‘on-plate’ detection for interactions of Xb22a and Xb22b isoforms with OsEREBP1 (AP2). Xb22a and Xb22b baits were expressed in pDEST32 (pDEST32/Xb22a and pDEST32/Xb22b, respectively) and OsEREBP1 prey was expressed in pDEST22 (pDEST22/AP2). Yeast strain MaV203 harboured the following combination of plasmids: 1a-4a) Four transformants of pDEST32/Xb22a + pDEST22/AP2; 1b-4b) Four transformants of pDEST32/Xb22b + pDEST22/AP2; 5) pDEST32 + pDEST22; 6) Positive control vectors pEXP32/Krev1 + pEXP22/RalGDS-wt; 7) pEXP32/Krev1 + pEXP22/RalGDS-m1; 8) pEXP32/Krev1 + pEXP22/RalGDS-m2; 9a) pDEST32/Xb22a + pDEST22; 9b) pDEST32/Xb22b + pDEST22; 10) pDEST32 + pDEST22/AP2. The Xb22a interacted strongly with OsEREBP1 as indicated by blue color in β-galactosidase assays for lacZ reporter gene (1a-4a). However, no color developed when Xb22b/pDEST32 was interacted with AP2/pDEST22 indicating that Xb22b does not interact with OsEREBP1 (1b-4b). The empty vector pDEST22 did not interact with Xb22a/pDEST32, Xb22b/pDEST32 and pDEST32 (9a, 9b) did not interact with and AP2/pDEST22 (10), whereas positive control (supplied with the kit) showed blue color (6,7,8). (TIF) [file pone.0127831.s001.tif]

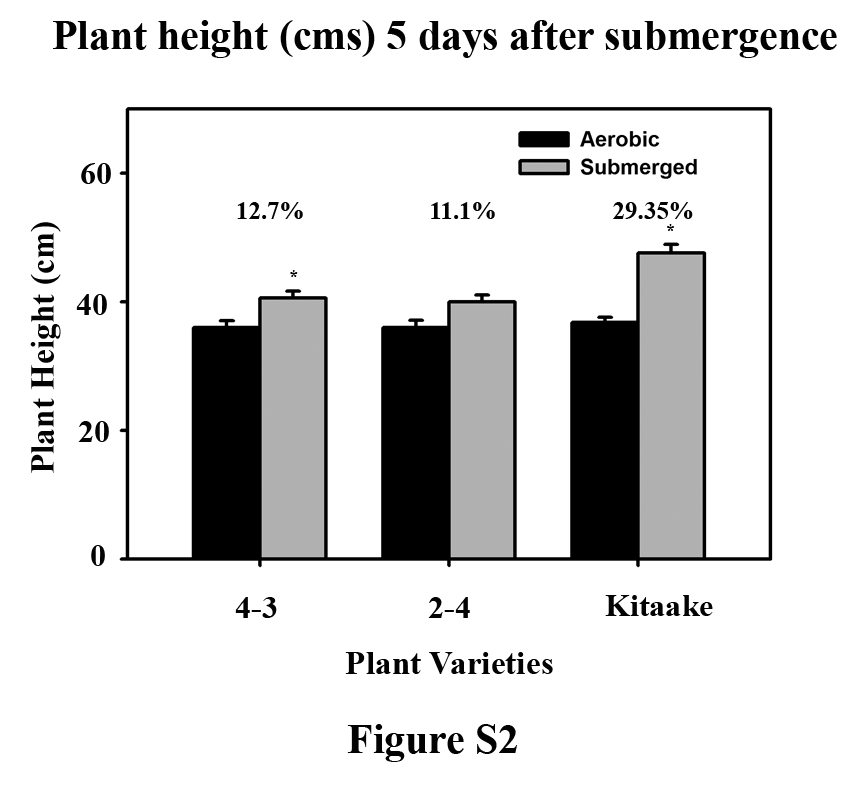

Supplement: S2 Fig — Two-week old plants were submerged for 5 days and the height of the plants was measured following submergence treatment. Plants grown under aerobic conditions were used as control. The error bars represent standard deviation of readings from 10 plants and asterisk indicates that the differences in length were significant (P<0.01). (TIF) [file pone.0127831.s002.tif]

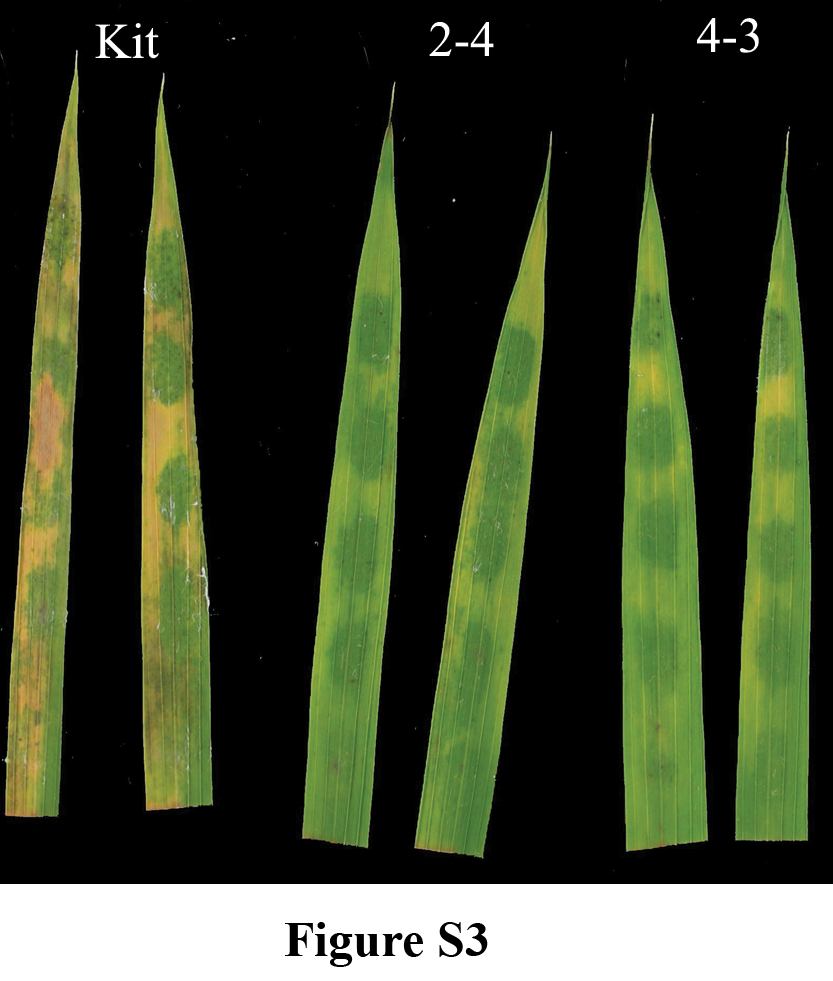

Supplement: S3 Fig — The latest fully expanded leaves from 3-week old control (Kit) and transgenic plants (2–4 and 4–3) were spotted with conidial spores (4x 10^4^ conidia/ml) suspended in 0.25% gelatin. The leaves were incubated in growth chamber on moist filter paper with 16h/8h of light/dark regime and were observed for lesion development. (TIF) [file pone.0127831.s003.tif]

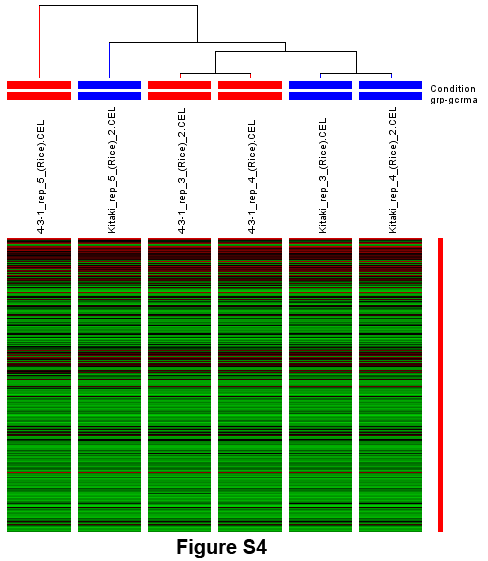

Supplement: S4 Fig — Of the three replicates of control (Kitaki) vs. sample (4-3-1), rep_3 and rep_4 of kitaki form one cluster, rep_3 and rep_4 of 4-3-1 form another cluster, whereas rep_5 of Kitaki and 4-3-1 are distinct. (TIF) [file pone.0127831.s004.tif]
