# Supplementary material for: Overexpression of an AP2/ERF Type Transcription Factor OsEREBP1 Confers Biotic and Abiotic Stress Tolerance in Rice
Source: PLoS One. 2015 Jun 2;10(6):e0127831. doi: 10.1371/journal.pone.0127831 (PMC4452794; doi:10.1371/journal.pone.0127831)
Supplement: S1 Table — (DOC) [file pone.0127831.s005.doc]

**Table S1.**

**List of primers used in this study**

| **Gene** | **Forward primer (5′–3′)** | **Reverse Primer (5′–3′)** |
| --- | --- | --- |
| *Jmt* | GTGGTGCCGAGCAATAATGCC | CACGTACATCCTCCCCTCGTT |
| *PR10* | CTTCATCGACGCCATTGAGGTC | CTTGAGCTTGCCCACCTTACTTC |
| *NAC6* | TCTCTACAAGTTCGACCCGTG | GCCTTCTTGATCCCAAGCGTG |
| *PBZ1* | GCAGGGAGCGTATACAAGACC | GTCTTCGAGCTCGTACTCCAC |
| *Wrky62* | TCCAAAGATGTGCGGAGGATAGG | CGACGAGTTGATGGAGATGGAG |
| *OsEREBP 1* | ACTGCCGGATTTGATGGTCCTG | CAGCATCATAAGCTCTTGCAGC |
| *L0X* | GTCGATGTCGTCAACCGCTTC | GACATCGTAGTCGTAGACGCG |
| *OsLiS* | GATCGATTGATTCGACCTGAATT | AATCTCTACACGCCATTATGCATG |
| *OsbHLH148* | ACGGAGATGAACGTCGAGAC | ATGAGATGTCTGTGTCCAGC |
| *LEA5* | CTCAGCCTTCTGGCACAAAG | TGGAGTTGGAGAGCAGCTTG |
| *CatB* | ATGGCCAGAGGATATCATCC | CTGGAGCAGCTTATCATCAGAG |
| *RERJ1* | CTGTCCGTGTCGTCCATCAG | TGATGTTGGCGGTGATGACG |
| *AP59* | GGCAACAAGCGGCCATATCC | GAACCAGTGCCATGGCCATG |
| *Actin* | CGGGAAATTGTGAGGGACAT | AGGAAGGCTGGAAGAGGACC |
| *Xb22a/b* | CACCATGGCGCTCGCCCACCAGCTG | TCATTTCTTGTTGAATCCAAAC |
| *OsEREBP1FL* | CACCATGTGCGGCGGCGCCATCATC | TCAATAGAAATCGCTAACGGGCAT |
| *OsEREBP1Smgfp* | CACCATGTGCGGCGGCGCCATCATC | ATAGAAATCGCTAACGGGCATGTC |
| *Xb22aSmgfp* | CACCATGGCGCTCGCCCACCAGCTG | TTTCTTGTTGAATCCAAAC |
| *HygS/AS* | ATGAAAAAGCCTGAACTCACCGCGA | TTCCTTTGCCCTCGGACGAGT |
| *UbiS/AP2AS* | TTGTCGATGCTCACCCTGTTGTTT | GAAGTTAGCAGATGGGTAGAC |
| *EF1a* | CAACAGTCGAAGGGCAATAATAAGTC | ACTGCCACACCTCCCACATTG |
